# The GCN5-CITED2-PKA signalling module controls hepatic glucose metabolism through a cAMP-induced substrate switch

**DOI:** 10.1038/ncomms13147

**Published:** 2016-11-22

**Authors:** Mashito Sakai, Tomoko Tujimura-Hayakawa, Takashi Yagi, Hiroyuki Yano, Masaru Mitsushima, Hiroyuki Unoki-Kubota, Yasushi Kaburagi, Hiroshi Inoue, Yoshiaki Kido, Masato Kasuga, Michihiro Matsumoto

**Affiliations:** 1 Department of Molecular Metabolic Regulation, Diabetes Research Center, Research Institute, National Center for Global Health and Medicine, 1-21-1 Toyama, Shinjuku-ku, Tokyo 162-8655, Japan; 2 Department of Bioregulation, Nippon Medical School, 1-396 Kosugi-machi, Nakahara-ku, Kawasaki 211-8533, Japan; 3 Department of Diabetic Complications, Diabetes Research Center, Research Institute, National Center for Global Health and Medicine, 1-21-1 Toyama, Shinjuku-ku, Tokyo 162-8655, Japan; 4 Metabolism and Nutrition Research Unit, Innovative Integrated Bio-research Core, Institute for Frontier Science Initiative, Kanazawa University, 13-1 Takara-machi, Kanazawa 920-8641, Japan; 5 Division of Medical Chemistry, Department of Metabolism and Disease, Kobe University Graduate School of Health Sciences, 7-10-2 Tomogaoka, Suma-ku, Kobe 654-0142, Japan; 6 Division of Diabetes and Endocrinology, Department of Internal Medicine, Kobe University Graduate School of Medicine, 7-5-1 Kusunoki-cho, Chuo-ku, Kobe 650-0017, Japan; 7 National Center for Global Health and Medicine, 1-21-1 Toyama, Shinjuku-ku, Tokyo 162-8655, Japan

## Abstract

Hepatic gluconeogenesis during fasting results from gluconeogenic gene activation via the glucagon–cAMP–protein kinase A (PKA) pathway, a process whose dysregulation underlies fasting hyperglycemia in diabetes. Such transcriptional activation requires epigenetic changes at promoters by mechanisms that have remained unclear. Here we show that GCN5 functions both as a histone acetyltransferase (HAT) to activate fasting gluconeogenesis and as an acetyltransferase for the transcriptional co-activator PGC-1α to inhibit gluconeogenesis in the fed state. During fasting, PKA phosphorylates GCN5 in a manner dependent on the transcriptional coregulator CITED2, thereby increasing its acetyltransferase activity for histone and attenuating that for PGC-1α. This substrate switch concomitantly promotes both epigenetic changes associated with transcriptional activation and PGC-1α–mediated coactivation, thereby triggering gluconeogenesis. The GCN5-CITED2-PKA signalling module and associated GCN5 substrate switch thus serve as a key driver of gluconeogenesis. Disruption of this module ameliorates hyperglycemia in obese diabetic animals, offering a potential therapeutic strategy for such conditions.

## Abstract (web summary)

GCN5 inhibits hepatic gluconeogenesis through acetylation of PGC-1α. Here the authors show that GCN5 also activates hepatic gluconeogenesis by acetylating histone H3K9, and that the affinity of GCN5 for its different substrates is regulated via phosphorylation at S275 by PKA in a CITED2-dependent manner.

Hepatic gluconeogenesis is induced by pancreatic glucagon during fasting to maintain glucose homeostasis^1^, a process whose dysregulation in diabetes results in fasting hyperglycemia^2^,^3^. The glucagon–cAMP–protein kinase A (PKA) pathway activates hepatic gluconeogenesis by inducing expression of the gluconeogenic genes *G6pc* (encoding the catalytic subunit of glucose-6-phosphatase) and *Pck1* (phosphoenolpyruvate carboxykinase)^4^. Such induction is thought to be mediated through orchestration of hormone-dependent epigenetic changes^5^ and assembly of transcriptional machinery^3^,^6^,^7^,^8^,^9^,^10^,^11^,^12^,^13^ at the gene promoters. A key component of this machinery is the CREB (cAMP response element-binding protein)–CRTC2 (CREB-regulated transcriptional co-activator 2) complex, whose assembly is triggered by PKA-dependent phosphorylation of CREB and the dephosphorylation of CRTC2 and which promotes recruitment of the histone acetyltransferase (HAT) CBP (CREB-binding protein, also known as Kat3a) and thereby activates peroxisome proliferator–activated receptor γ co-activator–1α (PGC-1α) gene transcription^6^. PGC-1α then functions with the transcription factors forkhead box O1 (FoxO1)^8^ and hepatocyte nuclear factor–4α (HNF-4α)^7^,^9^ to mediate synergistic activation of the gluconeogenic program; however, how glucagon signalling triggers epigenetic changes for efficient transcription of gluconeogenic genes has been unclear^5^.

General control non-repressed protein 5 (GCN5, also known as Kat2a) functions not only as a HAT that links histone acetylation and transcriptional activation^14^ but it also possesses acetyltransferase activity for non-histone substrates^15^,^16^. GCN5 thus acetylates and inactivates PGC-1α and thereby inhibits PGC-1α–dependent gluconeogenesis^17^,^18^,^19^. This function of GCN5 is inhibited during fasting by CBP- and p300-interacting transactivator with glutamic acid– and aspartic acid–rich COOH-terminal domain 2 (CITED2), a fasting-inducible transcriptional coregulator that binds to GCN5 and disrupts the GCN5–PGC-1α interaction, resulting in deacetylation and activation of PGC-1α (ref. 20); however, the precise role of GCN5 associated with CITED2 in the regulation of hepatic gluconeogenesis by the glucagon-cAMP-PKA axis has remained unknown.

We now show that GCN5 serves a dual function in the feeding-to-fasting transition: as a HAT to activate gluconeogenesis in the fasted state and as a PGC-1α acetyltransferase to inhibit it in the fed state. This dual function of GCN5 is reciprocally regulated by a glucagon-dependent substrate switch at the feeding-to-fasting transition. The fasting-induced GCN5-CITED2-PKA complex serves as a specific module for PKA-dependent phosphorylation of GCN5 at Ser^275^, which drives the substrate switch from PGC-1α to histone H3. This switch integrates epigenetic changes and the co-activator activity of PGC-1α, leading to full induction of the gluconeogenic program. Disruption of the GCN5-CITED2-PKA signalling module ameliorates hyperglycemia in obese diabetic animals and is therefore a potential strategy for the treatment of such conditions.

## Results

### Hepatic GCN5 expression is regulated by cAMP-PKA signalling

**Figure 1:**
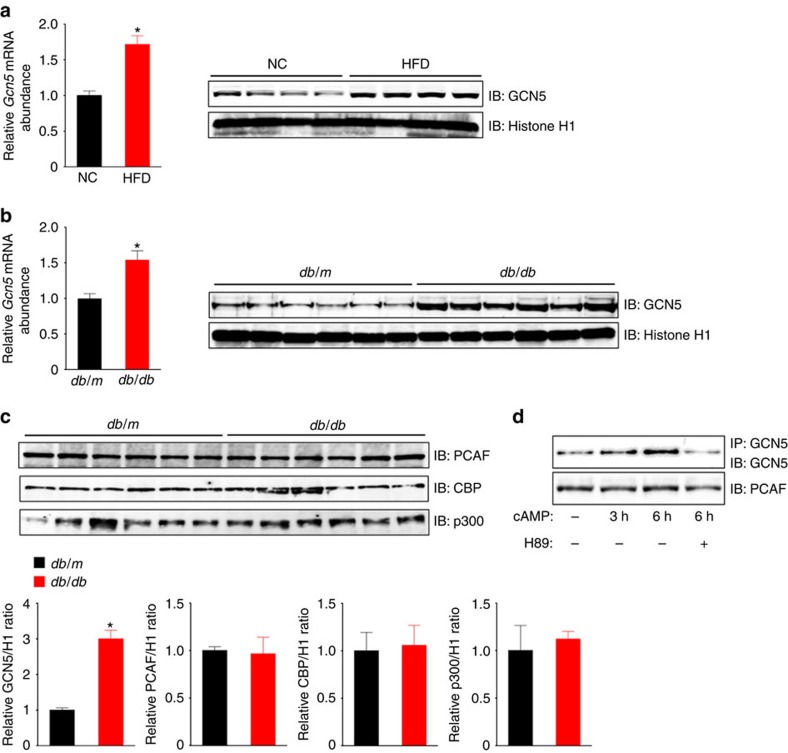
Hepatic expression of GCN5 is upregulated in obese diabetic mice via cAMP-PKA signalling. (**a**,**b**) qRT-PCR analysis of *Gcn5* mRNA and immunoblot (IB) analysis of nuclear GCN5 in the liver of C57BL/6J mice maintained on normal chow (NC) or a HFD (**a**) or of *db/db* or *db/m* (control) mice (**b**) after deprivation of food for 16 h. RT–PCR data are means±s.e.m. (*n*=7 (**a**) or 6 (**b**)). Histone H1 was examined as a loading control for immunoblot analysis. (**c**) Immunoblot analysis of GCN5, PCAF, CBP and p300 in the liver of *db/db* or *db/m* mice deprived of food for 16 h. Quantitative data are means±s.e.m. (*n*=3). (**d**) Primary mouse hepatocytes were incubated in the absence or presence of 100 μM pCPT-cAMP or the PKA inhibitor H89 (20 μM) for the indicated times. Cell lysates were then subjected to immunoblot analysis of PCAF or to IP followed by immunoblot analysis with antibodies to GCN5. Data are representative of at least three independent experiments. Statistical analysis was performed with the unpaired Student's *t*-test (**a**–**c**). *P<0.05 versus NC (**a**) or *db/m* mice (**b**,**c**). RT–PCR, PCR with reverse transcription.

We first investigated hepatic expression of GCN5 as well as of CBP, its paralog p300 (also known as Kat3b), and the GCN5 paralog p300/CBP-associated factor (PCAF, also known as Kat2b) in mice. Among these HATs, we found that the expression of GCN5 was selectively increased in the liver of two mouse models of obesity-associated type 2 diabetes—normal mice fed a high-fat diet (HFD) (Fig. 1a) and *db/db* mice (Fig. 1b,c)—compared with control mice. The amount of GCN5 in primary cultured mouse hepatocytes was also increased by treatment with a cell-permeable analogue of cAMP (pCPT-cAMP) in a PKA-dependent manner (Fig. 1d), suggesting that glucagon-cAMP-PKA signalling increases the hepatic abundance of GCN5 as well as that of CITED2 (ref. 20). These findings prompted us to examine whether GCN5 regulates gluconeogenesis through histone acetylation in collaboration with CITED2.

### GCN5 depletion attenuates gluconeogenesis *in vivo* and *in vitro*

**Figure 2:**
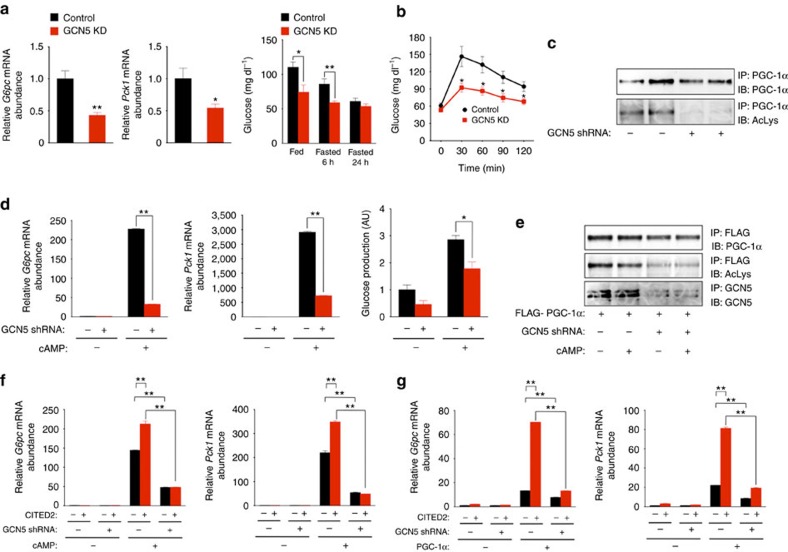
GCN5 depletion suppresses hepatic gluconeogenesis *in vivo* and *in vitro*. (**a**,**b**) Effects of shRNA-mediated knockdown (KD) of GCN5 in the liver of C57BL/6J mice on hepatic gluconeogenic gene expression under the fasted (24 h) condition (**a**) or on plasma glycemia either under fasted (6 or 24 h) or fed conditions (**a**) or after pyruvate administration (**b**). (**c**) IP and immunoblot analysis of acetylated (Ac) PGC-1α in the liver of C57BL/6J mice injected with an adenovirus for GCN5 shRNA and deprived of food for 24 h. (**d**) Effects of shRNA-mediated depletion of GCN5 on gluconeogenic gene expression and glucose production in primary mouse hepatocytes exposed (or not) to pCPT-cAMP for 16 h. (**e**) IP and immunoblot analysis of acetylated PGC-1α in primary hepatocytes expressing FLAG–PGC-1α with or without GCN5 depletion and incubated in the absence or presence of pCPT-cAMP for 6 h. (**f**) Effects of GCN5 depletion on CITED2-dependent enhancement of gluconeogenic gene expression induced by pCPT-cAMP (100 μM, 6 h) in primary hepatocytes. (**g**) Effects of GCN5 knockdown on PGC-1α-induced gluconeogenic gene expression with or without CITED2 overexpression in primary hepatocytes. All quantitative data are means±s.e.m. (*n*=7 (**a**,**b**) or 3 (**d**,**f**,**g**)). Statistical analysis was performed with the unpaired Student's *t*-test (**a**) or ANOVA followed by Bonferroni's *post hoc* test (**b**,**d**,**f**,**g**). *P<0.05, **P<0.01 compared with control or as indicated. Data in **c**,**e** are representative of at least three independent experiments. Adenoviral vectors encoding GCN5 shRNA, FLAG–PGC-1α or CITED2 were used for these experiments. ANOVA, analysis of variance.

In lean mice, depletion of hepatic GCN5 with a short hairpin RNA (shRNA) reduced the level of gluconeogenic gene expression apparent after food deprivation for 24 h as well as the blood glucose concentration either after fasting for 6 h (Fig. 2a and Supplementary Fig. 1a) or after administration of pyruvate (Fig. 2b) despite the associated marked attenuation of PGC-1α acetylation (Fig. 2c). Knockdown of hepatic GCN5 in *db/db* mice also reduced fasting gluconeogenic gene expression and glycemia (Supplementary Fig. 1b). In primary mouse hepatocytes, GCN5 knockdown attenuated gluconeogenic gene expression as well as glucose production induced by pCPT-cAMP (Fig. 2d and Supplementary Fig. 1c) despite the observed downregulation of PGC-1α acetylation (Fig. 2e). These results thus suggested that GCN5 contributes—in a manner independent of its PGC-1α acetyltransferase function—to gluconeogenic gene induction via the glucagon-cAMP pathway.

In hepatocytes, forced expression of CITED2 enhances gluconeogenic gene expression induced by cAMP (Fig. 2f and Supplementary Fig. 1d) or by PGC-1α overexpression in the absence of cAMP^20^ (Fig. 2g and Supplementary Fig. 1e). The PGC-1α acetyltransferase activity of GCN5 is suppressed by interaction with CITED2 under these conditions^20^. We therefore examined whether GCN5 that interacts with CITED2 might function other than as a PGC-1α acetyltransferase in the regulation of gluconeogenesis by adopting loss- and gain-of-function approaches in this setting. Loss of GCN5 attenuated the CITED2-dependent enhancement of gluconeogenic gene expression induced by cAMP (Fig. 2f and Supplementary Fig. 1d) or by PGC-1α overexpression (Fig. 2g and Supplementary Fig. 1e) despite the downregulation of PGC-1α acetylation induced by GCN5 knockdown (Fig. 2e).

### GCN5 promotes gluconeogenesis in concert with CITED2

**Figure 3:**
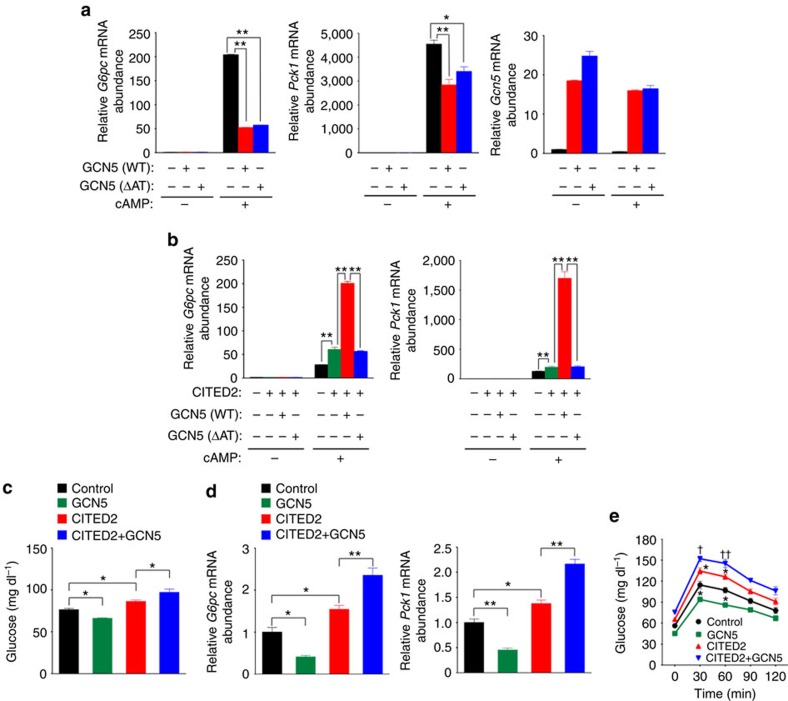
GCN5 promotes gluconeogenesis in an acetyltransferase- and CITED2-dependent manner. (**a**) Quantitative RT-PCR analysis of *Gcn5* and gluconeogenic gene expression in primary hepatocytes infected with adenoviruses for WT or ΔAT mutant forms of GCN5 and exposed to pCPT-cAMP for 6 h. (**b**) Effects of forced expression of WT or ΔAT forms of GCN5 together with CITED2 on pCPT-cAMP-induced gluconeogenic gene expression in primary hepatocytes. (**c**–**e**) Effects of GCN5 overexpression with or without that of CITED2 in the liver of C57BL/6J mice on glycemia under the fasted (6 h) condition (**c**) or after pyruvate administration (**e**) as well as on hepatic gluconeogenic gene expression under the fasted (24 h) condition (**d**). All data are means±s.e.m. (*n*=3 (**a**,**b**), 10 (**c**) or 8 (**d**,**e**)). Statistical analysis was performed with ANOVA followed by Bonferroni's *post hoc* test. *P<0.05, **P<0.01 compared with control or as indicated; †*P*<0.05, ††*P*<0.01 versus CITED2. Adenoviral vectors encoding GCN5(WT), GCN5(ΔAT) or CITED2 were used for these experiments. ANOVA, analysis of variance; RT–PCR, PCR with reverse transcription.

Overexpression of wild-type (WT) GCN5 suppressed gluconeogenic gene induction by cAMP in primary hepatocytes (Fig. 3a), consistent with the previously observed suppression of PGC-1α–induced gluconeogenic gene expression by GCN5 overexpression in Fao hepatoma cells^17^. This effect was also observed with a mutant (ΔAT) of GCN5 that lacks acetyltransferase activity^21^ toward both histone H3 and PGC-1α (Fig. 3a and Supplementary Fig. 2a,b), however, indicating that GCN5 suppresses gluconeogenic gene expression independently of its enzymatic activity in this setting.

In contrast, coexpression of GCN5(WT), but not that of GCN5(ΔAT), with CITED2 enhanced cAMP-induced gluconeogenic gene expression (Fig. 3b and Supplementary Fig. 2c). We also examined the effects of overexpression of GCN5, CITED2, and both proteins in the liver of C57BL/6J mice on gluconeogenesis. As previously shown^20^, mice overexpressing CITED2 in the liver manifested increased fasting glycemia (Fig. 3c) and hepatic expression of *G6pc* and *Pck1* (Fig. 3d and Supplementary Fig. 2d) compared with control mice. Hepatic overexpression of GCN5 alone reduced both fasting glycemia (Fig. 3c) and hepatic expression of gluconeogenic genes (Fig. 3d and Supplementary Fig. 2d), consistent with our *in vitro* results (Fig. 3a) as well as previous observations^17^. Again consistent with our *in vitro* findings (Fig. 3b), coexpression of GCN5 with CITED2 in the liver of C57BL/6J mice increased both fasting glycemia (Fig. 3c) and hepatic gluconeogenic gene expression (Fig. 3d and Supplementary Fig. 2d) compared with those apparent with CITED2 expression alone. To evaluate the effects of such altered gluconeogenic gene expression, we performed a pyruvate challenge test. Glucose levels after pyruvate administration were significantly higher in mice with hepatic CITED2 overexpression but were lower in those with hepatic GCN5 overexpression compared with control mice (Fig. 3e). Mice with hepatic coexpression of CITED2 and GCN5 showed blood glucose concentrations after pyruvate administration that were higher than those in mice with CITED2 overexpression alone (Fig. 3e). Together, these *in vitro* and *in vivo* results suggested that GCN5 might function as a cAMP-responsive, CITED2-dependent HAT in the regulation of gluconeogenesis.

### GCN5 is a crucial HAT for initiation of gluconeogenesis

**Figure 4:**
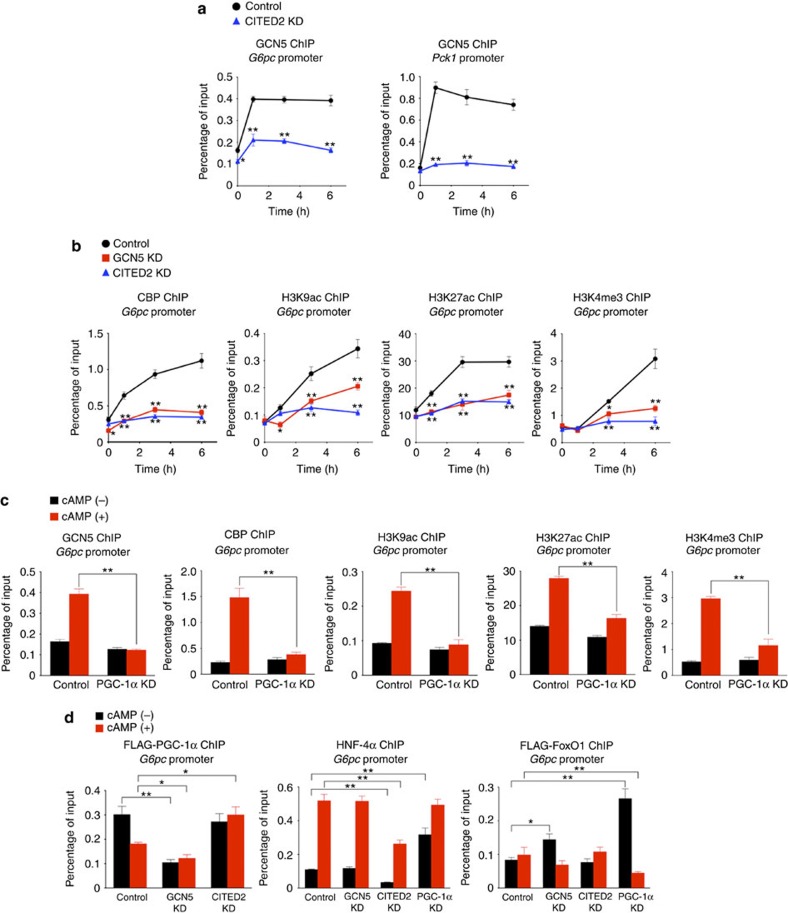
GCN5 recruitment to gluconeogenic gene promoters is regulated by cAMP in a CITED2- and PGC-1α-dependent manner in primary hepatocytes. (**a**) ChIP-qPCR analysis of the occupancy of *G6pc* and *Pck1* promoters with GCN5 in cells depleted of CITED2 and exposed to pCPT-cAMP for the indicated times. (**b**) ChIP-qPCR analysis of the occupancy of the *G6pc* promoter with CBP or epigenomic marks in cells depleted of GCN5 or CITED2 and exposed to pCPT-cAMP for the indicated times. (**c**) ChIP-qPCR analysis of the occupancy of the *G6pc* promoter with GCN5, CBP or epigenomic modifications in cells depleted of PGC-1α and exposed to pCPT-cAMP for 6 h. (**d**) ChIP-qPCR analysis of the occupancy of the *G6pc* promoter with FLAG-tagged PGC-1α, HNF-4α or FLAG-FoxO1 in cells depleted of GCN5, CITED2 or PGC-1α and exposed to pCPT-cAMP for 6 h. All data are means±s.e.m. (*n*=3). *P<0.05, **P<0.01 versus control or as indicated (ANOVA with Bonferroni's *post hoc* test). Adenoviral vectors encoding GCN5, CITED2 or PGC-1α shRNAs, FLAG–PGC-1α or FLAG-FoxO1 were used for these experiments. ANOVA, analysis of variance; qPCR, quantitative PCR.

To test this hypothesis, we examined the molecular signatures of gluconeogenic gene promoters by chromatin immunoprecipitation (ChIP) and quantitative PCR analysis in primary hepatocytes depleted of GCN5, CITED2 or PGC-1α. The occupancy of such promoters with GCN5 and CBP was increased after exposure of control hepatocytes to pCPT-cAMP for 1–6 h (Fig. 4a,b and Supplementary Fig. 3a). The amounts of histone H3 acetylated at Lys^9^ (H3K9ac) or Lys^22^ (H3K27ac)—which are generated predominantly by GCN5 and CBP, respectively^22^—as well as that of trimethylated H3K4 (H3K4me3) (ref. 23), all three of which marks are associated with active gene transcription^24^, were also increased at these promoters in cells treated with pCPT-cAMP (Fig. 4b and Supplementary Fig. 3a). Consistent with the associated downregulation of gluconeogenic gene transcription^20^ (Fig. 2d), these cAMP-induced increases in the amounts of H3K9ac, H3K27ac and H3K4me3 were blunted by depletion of either GCN5 (Fig. 4b and Supplementary Fig. 3a,e), CITED2 (Fig. 4b and Supplementary Fig. 3a,e) or PGC-1α (Fig. 4c and Supplementary Fig. 3b,e). The cAMP-induced recruitment of GCN5 and CBP was also attenuated by depletion of either CITED2 (Fig. 4a,b and Supplementary Fig. 3a,e) or PGC-1α (Fig. 4c and Supplementary Fig. 3b,e). In addition, the cAMP-induced recruitment of CBP to gluconeogenic gene promoters was impaired by GCN5 depletion (Fig. 4b and Supplementary Fig. 3a,e), indicating that GCN5 is required for the recruitment of CBP. On the other hand, whereas the increased occupancy of gluconeogenic gene promoters with PGC-1α or FoxO1 observed in immortalized murine hepatocytes treated with forskolin and dexamethasone^8^ or in primary mouse hepatocytes exposed to glucagon for 1 h (ref. 11), respectively, was not apparent in primary cultured mouse hepatocytes after exposure to pCPT-cAMP for 6 h, promoter occupancy with PGC-1α, FoxO1, or HNF-4α was also dysregulated by depletion of GCN5, CITED2 or PGC-1α—although we did not identify a unifying defect in the binding of these regulators that might account for the impaired induction of gluconeogenic genes (Fig. 4d and Supplementary Fig. 3c,e).

To clarify whether epigenetic changes induced by GCN5 depletion are restricted to gluconeogenic gene promoters or are more widespread, we examined the effects of GCN5 knockdown on the epigenetic status of the promoters of two nongluconeogenic genes: *Gapdh* (encoding glyceraldehyde-3-phosphate dehydrogenase) and *Hmgcr* (hydroxymethylglutarate–coenzyme A reductase). Depletion of GCN5 affected neither acetylation of H3K9 or H3K27 or trimethylation of H3K4 at these promoters nor the corresponding mRNA abundance (Supplementary Fig. 3d), indicating that the epigenetic changes associated with GCN5 knockdown occur selectively at gluconeogenic gene promoters. We also investigated whether these epigenetic changes induced by GCN5 depletion are observed in mouse liver. Consistent with our *in vitro* results, the fasting levels of H3K9 and H3K27 acetylation (but not that of H3K4 trimethylation) at the gluconeogenic gene promoters were reduced by GCN5 depletion (Supplementary Fig. 3f). The difference in the effect of GCN5 depletion on H3K4 trimethylation status between the *in vivo* and *in vitro* results may reflect a non-cell-autonomous response to compensate for the impairment of gluconeogenic gene expression induced by hepatic depletion of GCN5. Collectively, these data indicated that GCN5 is a crucial HAT for the initiation of cAMP-induced gluconeogenic gene transcription in concert with CITED2.

### cAMP and CITED2 promote a GCN5 substrate switch

**Figure 5:**
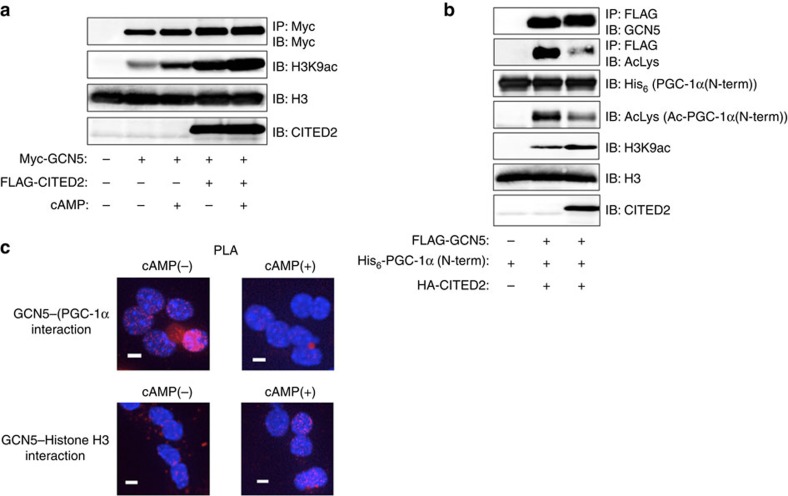
GCN5 switches substrate in a cAMP- and CITED2-dependent manner. (**a**) Immunoblot analysis of the effects of FLAG-CITED2 expression or pCPT-cAMP treatment (for 1 h) in AML12 cells on the HAT activity of immunoprecipitated Myc epitope-tagged GCN5 assayed *in vitro* with histone H3 as substrate. (**b**) Effects of haemagglutinin epitope (HA)-tagged CITED2 expression in AML12 cells on the acetyltransferase activity of immunoprecipitated FLAG-GCN5 assayed *in vitro* with histone H3 and a His_6_-tagged NH_2_-terminal fragment of PGC-1α as substrates. (**c**) Interaction of GCN5 with PGC-1α or histone H3 was assessed by PLA in primary hepatocytes expressing either Myc-GCN5 with FLAG–PGC-1α (top) or FLAG-GCN5 alone (bottom) and exposed (or not) to pCPT-cAMP for 1 h. PLA signals (red dots) represent proximity (<40 nm) of GCN5 and either PGC-1α (top) or histone H3 (bottom). Nuclei are stained blue with 4′,6-diamidino-2-phenylindole. Scale bars, 10 μm. All data are representative of at least three independent experiments. Adenoviral vectors were used for these experiments.

Given that glucagon-induced GCN5-CITED2 interaction is mediated by the cAMP-PKA pathway^20^, we tested whether GCN5 activity is regulated by cAMP, CITED2 or both in hepatocytes. The HAT activity of GCN5 measured *in vitro* with histone H3 as a substrate was increased by treatment of AML12 cells with pCPT-cAMP, and this effect was enhanced by CITED2 overexpression in an additive manner (Fig. 5a). These data thus indicated that the HAT activity of GCN5 was increased in the presence of cAMP and CITED2. We next examined the effect of CITED2 on the balance between the HAT and PGC-1α acetyltransferase activities of GCN5 measured *in vitro* with histone H3 and an NH_2_-terminal fragment of PGC-1α as substrates. In this setting, GCN5 activity was reciprocally regulated by CITED2: HAT activity was increased, whereas PGC-1α acetyltransferase activity and autoacetylation activity were decreased (Fig. 5b). These results indicated that CITED2 binds to GCN5 and promotes a substrate switch from PGC-1α to histone H3 as measured *in vitro*. To examine whether this switch also occurs in hepatocytes, we investigated the interaction of GCN5 with PGC-1α or histone H3 in the nucleus with an *in situ* proximity ligation assay (PLA)^25^. Exposure of primary hepatocytes to pCPT-cAMP disrupted GCN5–PGC-1α interaction and promoted GCN5–histone H3 interaction (Fig. 5c). Together with our results showing that GCN5 recruitment to and H3K9ac marking of gluconeogenic gene promoters are dependent on cAMP and CITED2 (Fig. 4a,b and Supplementary Fig. 3a), these findings supported the notion that a cAMP- and CITED2-dependent substrate switch of GCN5 integrates epigenetic changes and co-activator activity in gluconeogenic gene induction.

### PKA phosphorylates GCN5 within a module harboring CITED2

**Figure 6:**
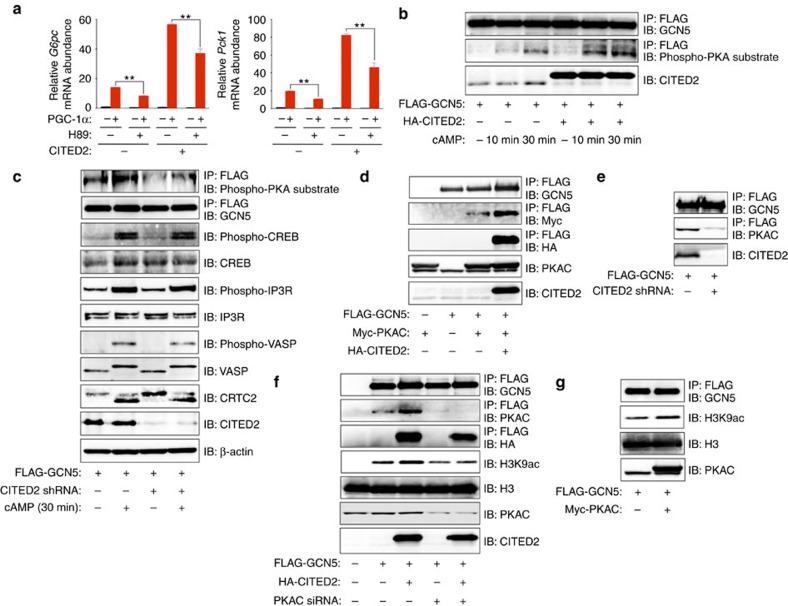
GCN5 is phosphorylated by PKA within a GCN5-CITED2-PKA signalling module. (**a**) Effects of PKA inhibition with H89 (20 μM, 6 h) on gluconeogenic gene expression induced by overexpression of PGC-1α with or without CITED2 in primary hepatocytes. Data are means±s.e.m. (*n*=3). **P<0.01 (ANOVA with Bonferroni's *post hoc* test). (**b**) Immunoblot analysis of the effects of HA-CITED2 expression or pCPT-cAMP treatment (10 or 30 min) on phosphorylation of FLAG-GCN5 in AML12 cells as assessed with antibodies to phosphorylated PKA substrates. (**c**) Effects of CITED2 depletion on pCPT-cAMP-induced phosphorylation of FLAG-GCN5 and other PKA substrates as well as on the dephosphorylation of CRTC2 in primary hepatocytes. (**d**,**e**) IP and immunoblot analysis of the interaction of FLAG-GCN5 with Myc-PKAC and HA-CITED2 (**d**) as well as of the effect of CITED2 knockdown on the interaction of FLAG-GCN5 with PKAC (**e**) in AML12 cells. (**f**) Effect of siRNA-mediated PKAC depletion in AML12 cells on basal and CITED2-induced HAT activity of FLAG-GCN5 as assessed by *in vitro* assay. (**g**) Effect of PKAC overexpression in AML12 cells on HAT activity of FLAG-GCN5 measured *in vitro*. A Myc-PKAC plasmid and PKAC siRNA were introduced into cells by transfection, whereas adenoviral vectors were used to introduce other exogenous proteins or shRNAs in these experiments. Data are representative of at least three independent experiments. ANOVA, analysis of variance; siRNA; small interfering RNA.

Given that recombinant CITED2 interacted with, but did not enhance the HAT activity of, GCN5 immunoprecipitated from AML12 cells overexpressing GCN5 alone (Supplementary Fig. 4a,b), we concluded that an additional factor that interacts with CITED2 might be necessary for the substrate switch. We found that, in the absence of pCPT-cAMP, inhibition of PKA by H89 significantly suppressed gluconeogenic gene induction by overexpression of PGC-1α alone or in combination with CITED2 in primary hepatocytes (Fig. 6a and Supplementary Fig. 4c), indicative of the requirement for PKA activity in this setting. PKA activated by glucagon-cAMP signalling mediates induction of gluconeogenesis and remodelling of the actin cytoskeleton through phosphorylation of CREB^26^,^27^ and the inositol 1,4,5-trisphosphate receptor (IP3R)^12^ and through that of vasodilator-stimulated phosphoprotein (VASP)^28^, respectively. We then tested whether GCN5 activity is regulated by PKA-mediated phosphorylation. With the use of antibodies specific for phosphorylated PKA substrates, we found that GCN5 phosphorylation was induced by pCPT-cAMP in AML12 cells (Fig. 6b) as well as by glucagon in mouse liver (Supplementary Fig. 4d). This effect of pCPT-cAMP was enhanced by CITED2 overexpression (Fig. 6b) and suppressed by shRNA-mediated CITED2 knockdown (Fig. 6c). In contrast, CITED2 knockdown did not affect either the phosphorylation of other PKA substrates such as CREB, IP3R, and VASP or the dephosphorylation of CRTC2 (as assessed on the basis of the associated band mobility shift) induced by pCPT-cAMP (Fig. 6c). These results suggested that PKA selectively phosphorylates GCN5 in a CITED2-dependent manner, prompting us to examine whether PKA interacts with the GCN5-CITED2 complex.

The PKA holoenzyme consists of two catalytic (PKAC) and two regulatory (PKAR) subunits, the latter of which serve as receptors for cAMP. The binding of cAMP to the regulatory subunits triggers dissociation and activation of the catalytic subunits^29^. Co-IP analysis of AML12 cells revealed CITED2-PKA interaction (Supplementary Fig. 4e) as well as CITED2-dependent GCN5-PKA interaction (Fig. 6d,e) at the level of endogenous or epitope-tagged proteins, indicative of formation of a GCN5-CITED2-PKA complex. We also tested whether PKAC alters the HAT activity of GCN5. Knockdown of PKAC almost completely attenuated the CITED2-induced increase in HAT activity (Fig. 6f), whereas forced expression of PKAC alone upregulated this activity (Fig. 6g). Together, these results indicated that GCN5, CITED2 and PKA form a complex that functions as a module for PKA-dependent upregulation of the HAT activity of GCN5. Co-IP analysis also revealed that the interaction of GCN5 with PKA or CITED2 (Supplementary Fig. 4f) as well as that of CITED2 with PKA (Supplementary Fig. 4g) were disrupted by exposure of AML12 cells to pCPT-cAMP for 30 min. PKA-dependent phosphorylation of GCN5 was also observed concomitantly with these effects (Supplementary Fig. 4h), and cAMP-induced disruption of the CITED2-GCN5 interaction was blocked by PKA inhibition (Supplementary Fig. 4i). Together, these results suggested that the GCN5-CITED2-PKA signalling module disassembles in response to phosphorylation of GCN5 by cAMP-activated PKA.

### GCN5 phosphorylation at Ser^275^ drives the substrate switch

**Figure 7:**
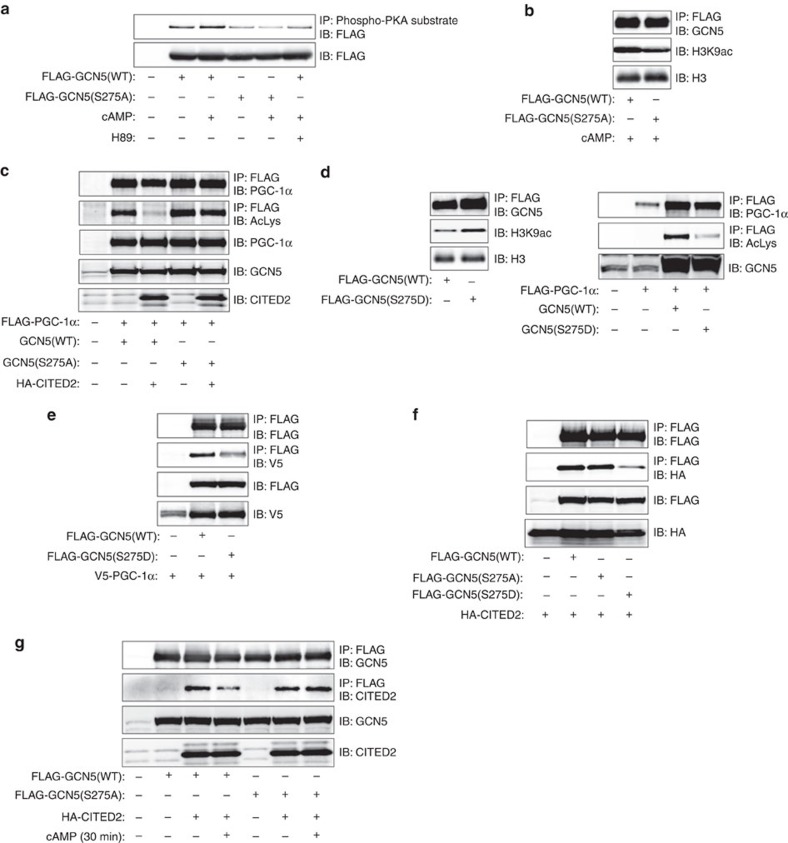
GCN5 phosphorylation at Ser^275^ drives its substrate switch. (**a**) AML12 cells expressing FLAG-tagged GCN5(WT) or GCN5(S275A) were exposed to pCPT-cAMP with or without H89 for 30 min and then subjected to IP with antibodies to phosphorylated PKA substrates followed by immunoblot analysis with antibodies to FLAG. (**b**) Effect of the S275A mutation of FLAG-GCN5 on *in vitro* acetylation of histone H3 in AML12 cells treated with pCPT-cAMP (1 h). (**c**) Acetylation of FLAG–PGC-1α in AML12 cells expressing GCN5(WT) or GCN5(S275A) with or without HA-CITED2. (**d**) Effects of the S275D mutation of GCN5 on histone H3 and PGC-1α acetyltransferase activities in AML12 cells. (**e**) Effect of the S275D mutation of FLAG-GCN5 on interaction with V5-tagged PGC-1α in AML12 cells. (**f**) Effects of the S275A and S275D mutations of FLAG-GCN5 on interaction with HA-CITED2 in AML12 cells. (**g**) The S275A mutation of GCN5 blocks the pCPT-cAMP-induced dissociation of HA-CITED2 from FLAG-GCN5 in AML12 cells. All data are representative of at least three independent experiments. Adenoviral vectors were used for these experiments.

We next explored further the role of PKA-dependent phosphorylation in the regulation of GCN5 action within the module. *In silico* analysis identified five serine or threonine residues conserved between rodents and human as putative PKA phosphorylation sites in GCN5 (Supplementary Fig. 5a). We therefore generated a series of putative phosphorylation-defective mutants, in which these residues are replaced with alanine. Similar to PKA inhibition with H89, the S275A mutation abolished basal and cAMP-induced phosphorylation of GCN5 as assessed with the antibodies to phosphorylated PKA substrates (Fig. 7a). The phosphorylation signal intensity for GCN5(S275A) was similar to that for GCN5(WT) in cells exposed to both cAMP and H89 and may therefore be attributable to phosphorylation at a PKA-independent site detectable by these antibodies. Immunoblot analysis of immunoprecipitates prepared from primary hepatocytes with antibodies to the Ser^275^-phosphorylated form of GCN5 also confirmed that pCPT-cAMP induced the phosphorylation of GCN5 in a PKA- and CITED2-dependent manner (Supplementary Fig. 5b). In addition, recombinant PKAC phosphorylated GCN5(WT), but not GCN5(S275A), *in vitro*, suggesting that PKA directly phosphorylates Ser^275^ of GCN5 (Supplementary Fig. 5c). Collectively, these results indicated that Ser^275^ is the principal PKA phosphorylation site in GCN5.

We examined whether phosphorylation of GCN5 at Ser^275^ regulates acetyltransferase activity with the use of the S275A mutant and the phosphorylation-mimicking mutant S275D. GCN5(S275A) manifested reduced HAT activity (Fig. 7b) and similar PGC-1α acetyltransferase activity (Supplementary Fig. 5d) compared with the WT protein, but it was resistant to CITED2-induced suppression of PGC-1α acetyltransferase activity in AML12 cells (Fig. 7c). In contrast, GCN5(S275D) showed increased HAT activity, reduced PGC-1α acetyltransferase activity, and an attenuated interaction with PGC-1α (Fig. 7d,e). These results suggested that PKA-dependent phosphorylation of GCN5 at Ser^275^ drives its substrate switch.

We also tested whether GCN5 phosphorylation at Ser^275^ affects its interaction with CITED2. The amount of HA-tagged CITED2 that co-immunoprecipitated from AML12 cells with FLAG-tagged GCN5 was reduced for the S275D mutant compared with the WT or S275A forms (Fig. 7f). Exposure of cells to pCPT-cAMP also reduced the amount of HA-CITED2 that co-immunoprecipitated with GCN5(WT) but not of that co-immunoprecipitating with GCN5(S275A) (Fig. 7g). Phosphorylation of GCN5 at Ser^275^ thus appears to promote disassembly of the GCN5-CITED2-PKA signalling complex, supporting a role for this module in phosphorylation of GCN5 at this site by PKA.

### Phosphorylation of GCN5 at Ser^275^ promotes gluconeogenesis

**Figure 8:**
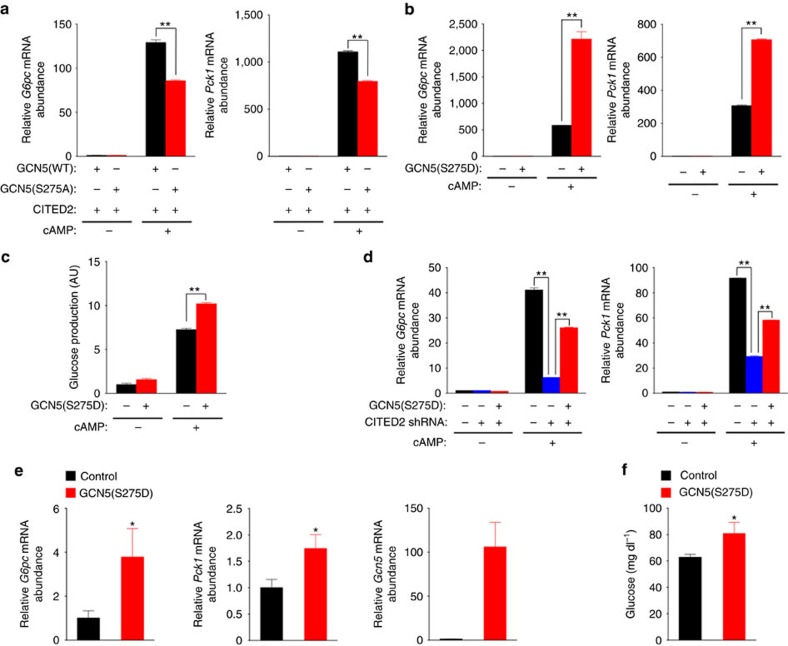
Phosphorylation of GCN5 at Ser^275^ promotes gluconeogenesis. (**a**) Effects of forced expression of GCN5(WT) or GCN5(S275A) together with CITED2 on gluconeogenic gene expression in primary hepatocytes exposed (or not) to pCPT-cAMP (6 h). (**b**,**c**) Effects of forced expression of GCN5(S275D) on gluconeogenic gene expression (**b**) and glucose production (**c**) in primary hepatocytes exposed (or not) to pCPT-cAMP (16 h). (**d**) qRT-PCR analysis of *G6pc* and *Pck1* expression in primary mouse hepatocytes infected with adenoviruses encoding CITED2 shRNA or GCN5(S275D) and exposed (or not) to pCPT-cAMP for 6 h. (**e**,**f**) Effects of GCN5(S275D) expression in the liver of C57BL/6J mice on gluconeogenic gene expression (**e**) and plasma glycemia (**f**) under the fasted (24 h) condition. All data are means±s.e.m. (*n*=3 (**a**–**d**) or 7 (**e**,**f**)). *P<0.05, **P<0.01 versus control or as indicated (ANOVA with Bonferroni's *post hoc* test (**a**–**d**) or unpaired Student's *t*-test (**e**,**f**)). Adenoviral vectors were used for these experiments. RT–PCR, PCR with reverse transcription. ANOVA, analysis of variance.

We next investigated the effects of these GCN5 mutants on gluconeogenesis *in vitro*. In primary hepatocytes overexpressing CITED2, pCPT-cAMP-induced gluconeogenic gene expression was enhanced to a lesser extent by GCN5(S275A) than by GCN5(WT) (Fig. 8a and Supplementary Fig. 6a). On the other hand, expression of the S275D mutant without CITED2 overexpression enhanced cAMP-induced gluconeogenic gene expression (Fig. 8b and Supplementary Fig. 6b)—in contrast to the effect of GCN5(WT) (Fig. 3a)—resulting in enhanced glucose production (Fig. 8c). The selective gluconeogenic effect of GCN5(S275D) was associated with enhanced cAMP-induced acetylation of H3K9 and H3K27 at gluconeogenic gene promoters, but not at *Gapdh* or *Hmgcr* promoters (Supplementary Fig. 6c). GCN5(S275D) also rescued, at least in part, the inhibition of gluconeogenic gene expression by CITED2 depletion in pCPT-cAMP-stimulated hepatocytes (Fig. 8d and Supplementary Fig. 6d), suggesting that GCN5 phosphorylated at Ser^275^ functions as a gluconeogenic HAT downstream of CITED2. These results indicated that phosphorylation of GCN5 at Ser^275^ by PKA in the GCN5-CITED2-PKA signalling module drives the substrate switch of GCN5 and thereby promotes gluconeogenesis *in vitro*.

We also assessed the phosphorylation level of hepatic GCN5 at Ser^275^ in a physiological setting *in vivo*. The phosphorylation level of GCN5 at Ser^275^ in the liver of lean mice was increased in the fasted state compared with the fed state (Supplementary Fig. 6e). To confirm the physiological relevance of this finding, we examined the time courses of GCN5 phosphorylation, PGC-1α acetylation, GCN5-CITED2 interaction and gluconeogenic gene expression in the liver of lean mice expressing both FLAG-GCN5 and HA-CITED2 at near physiological levels (about twice the abundance of the endogenous proteins) during fasting after refeeding for 12 h subsequent to a previous overnight fast. The initial increase in GCN5-CITED2 interaction (assessed on the basis of the amount of CITED2 co-immunoprecipitated with GCN5) was followed by increases in the level of GCN5 phosphorylation at Ser^275^ and in PGC-1α abundance (Supplementary Fig. 6f). Furthermore, the increase in the level of GCN5 phosphorylation occurred concomitantly with a reduction in the level of PGC-1α acetylation and with the maximal upregulation of gluconeogenic gene expression (Supplementary Fig. 6f). In addition, expression of GCN5(S275D) in the liver of lean mice increased gluconeogenic gene expression (Fig. 8e) and blood glucose levels (Fig. 8f) in the fasted condition. These data thus suggested that phosphorylation of GCN5 at Ser^275^ within the GCN5-CITED2-PKA signalling module during fasting drives the substrate switch of GCN5, resulting in activation of PGC-1α and of the HAT function of GCN5 and consequent promotion of gluconeogenesis.

### Suppression of GCN5 phosphorylation ameliorates diabetes

**Figure 9:**
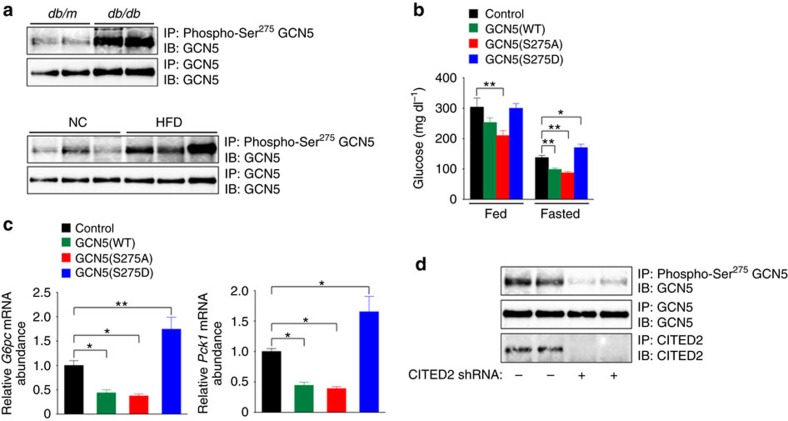
Suppression of hepatic GCN5 phosphorylation at Ser^275^ ameliorates diabetes. (**a**) Analysis of GCN5 phosphorylated at Ser^275^ in the liver of *db/db* or *db/m* (control) mice or of C57BL/6J mice fed NC or a HFD. All mice were deprived of food for 16 h before analysis. Liver extracts were subjected to IP with antibodies to Ser^275^-phosphorylated GCN5 followed by immunoblot analysis with antibodies to GCN5. (**b**,**c**) Effects of expression of GCN5(WT), GCN5(S275A) or GCN5(S275D) in the liver of *db/db* mice on plasma glucose concentration under fed or fasted (24 h) conditions (**b**) as well as on hepatic expression of *G6pc* and *Pck1* under the fasted (24 h) condition (**c**). (**d**) Effect of CITED2 depletion on GCN5 phosphorylation at Ser^275^ in the liver of *db/db* mice deprived of food for 24 h. All quantitative data are means±s.e.m. (*n*=7 (**b**,**c**)). *P<0.05, **P<0.01 (ANOVA with Bonferroni's *post hoc* test). Data in **a**,**d** are representative of at least three independent experiments. Adenoviral vectors were used for these experiments. ANOVA, analysis of variance; NC, normal chow.

We also examined the phosphorylation level of GCN5 at Ser^275^ in the liver of diabetic mice. Phosphorylation of GCN5 (Fig. 9a) as well as the amount of GCN5 protein (Fig. 1a–c) were increased in the liver of *db/db* mice and of C57BL/6J mice fed a HFD. The expression of CITED2 (ref. 20), but not that of PKA (Supplementary Fig. 7a), was also increased in the liver of diabetic mice. These changes in GCN5 and CITED2 are consistent with enhancement of gluconeogenesis and prompted us to address whether GCN5 phosphorylation at Ser^275^ contributes to hyperglycemia through promotion of hepatic gluconeogenesis in these animals. Hepatic expression of GCN5(S275D) increased, whereas that of GCN5(S275A) or GCN5(WT) decreased, blood glucose levels and gluconeogenic gene expression in the liver of fasted *db/db* mice (Fig. 9b,c and Supplementary Fig. 7b). These effects are consistent with those observed in hepatocytes (Fig. 8a–c) or in the liver of lean mice (Fig. 8e,f), and they indicate that inhibition of GCN5 phosphorylation at Ser^275^ suppresses gluconeogenesis and therefore ameliorates hyperglycemia, whereas enhanced phosphorylation of GCN5 at Ser^275^ promotes gluconeogenesis and exacerbates hyperglycemia, in diabetic animals. We have previously shown that depletion of CITED2 in the liver of *db/db* mice suppresses gluconeogenesis through enhancement of GCN5-dependent acetylation and the consequent inactivation of PGC-1α and thereby ameliorates diabetes^20^. In this setting, phosphorylation of GCN5 at Ser^275^ was also inhibited (Fig. 9d), indicating that disruption of the GCN5-CITED2-PKA signalling module by CITED2 depletion attenuates gluconeogenesis and ameliorates hyperglycemia through suppression of GCN5 phosphorylation at Ser^275^ and consequent enhanced GCN5-dependent acetylation and inhibition of PGC-1α as well as reduced GCN5-dependent acetylation of histone H3K9.

## Discussion

**Figure 10:**
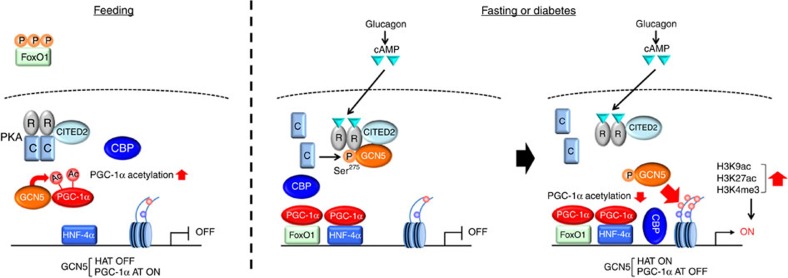
Model for regulation of hepatic gluconeogenesis by the GCN5-CITED2-PKA signalling module through a cAMP-induced substrate switch of GCN5. (left) In the fed state, the GCN5-CITED2-PKA signalling module is not assembled as a result of the low level of CITED2 expression maintained in the absence of glucagon signalling and the insulin-induced inhibition of GCN5-CITED2 interaction. GCN5 is not phosphorylated at Ser^275^ and therefore acetylates PGC-1α rather than histone H3, resulting in inactivation of PGC-1α and consequent suppression of gluconeogenic gene transcription. (middle) In the fasted state or diabetes, glucagon-cAMP signalling increases the expression of CITED2 and GCN5 and thereby promotes formation of the GCN5-CITED2-PKA signalling module, within which PKA activated by cAMP phosphorylates GCN5 at Ser^275^. (right) Phosphorylation of GCN5 induces a substrate switch from PGC-1α to histone H3 and a consequent increase in H3K9 acetylation and the deacetylation-mediated activation of PGC-1α at gluconeogenic gene promoters. The increase in the amount of H3K9ac promotes further epigenetic changes and the recruitment of transcriptional regulators required for initiation of gene transcription, whereas activated PGC-1α functions as a co-activator for gluconeogenic transcription factors such as FoxO1 and HNF-4α. These two effects act cooperatively to activate the gluconeogenic program. AT, acetyltransferase.

Our data show that, in the fasted state, glucagon-cAMP signalling increases the expression of CITED2 (ref. 20) and GCN5 as well as promotes the formation of a GCN5-CITED2-PKA signalling module. Within this module, PKA activated by cAMP phosphorylates GCN5 at Ser^275^ (with the phosphorylation of other PKA substrates such as CREB, VASP and IP3R being unaffected), resulting in a switch in the substrate preference of GCN5 from PGC-1α to histone H3 and a consequent increase in H3K9 acetylation and the deacetylation-mediated activation of PGC-1α at gluconeogenic gene promoters. The increase in the amount of H3K9ac promotes further epigenetic changes and the recruitment of transcriptional regulators required for initiation of gene transcription, whereas the activated PGC-1α functions as a co-activator for gluconeogenic transcription factors such as FoxO1 and HNF-4α. These two effects act cooperatively to activate the gluconeogenic program (Fig. 10). In the fed state, attenuation of glucagon signalling leads to downregulation of CITED2 and GCN5 expression, whereas insulin signalling inhibits GCN5-CITED2 interaction^20^, resulting in disruption of the GCN5-CITED2-PKA signalling module. In this condition, phosphorylation of GCN5 at Ser^275^ is inhibited and GCN5 therefore acetylates PGC-1α rather than histone H3, resulting in inactivation of PGC-1α and the consequent suppression of gluconeogenic gene transcription.

A previous study showed that GCN5 acetylates and suppresses the coactivation activity of PGC-1α (ref. 17). Overexpression of GCN5 in Fao cells thus suppressed gluconeogenic gene expression and glucose production induced by overexpression of PGC-1α (ref. 17). Overexpression of GCN5 in the liver of mice also reduced hepatic gluconeogenic gene expression in the fasted state as well as glycemia after fasting or pyruvate administration^17^ (Fig. 3c–e). In addition, the PGC-1α acetyltransferase function of GCN5 was previously shown to be activated by Sirt6 or the CDK4–cyclin D1 complex, whose expression is downregulated by glucagon-cAMP signalling or upregulated by insulin–GSK3β (glycogen synthase kinase 3β) signalling, respectively^18^,^19^. GCN5 has therefore been thought to function as a negative regulator of PGC-1α and, consequently, of hepatic gluconeogenesis (presumably in the fed state)^18^,^19^,^20^,^30^,^31^. On the other hand, the role of GCN5 in gluconeogenic gene induction by glucagon-cAMP signalling during fasting and its role as a HAT have remained to be elucidated. Given that CITED2 forms a complex with GCN5 during fasting and activates PGC-1α through inhibition of GCN5-dependent acetylation^20^, we focused on the fasting-inducible GCN5-CITED2 complex. Our data now provide evidence that GCN5 functions as a HAT to activate the gluconeogenic program in the fasted state in addition to as a PGC-1α acetyltransferase to inhibit it in the fed state. This dual function of GCN5 is reciprocally regulated by a glucagon-dependent substrate switch at the fed-to-fasting transition. The fasting-induced formation of the GCN5-CITED2-PKA complex allows the specific PKA-dependent phosphorylation of GCN5 at Ser^275^, which, in turn, drives the substrate switch of GCN5. This newly uncovered mechanism thus incorporates GCN5-mediated repression of the transcriptional co-activator activity of PGC-1α in the fed state as well as cAMP- and CITED2-mediated upregulation of such activity in the fasted state, and it therefore integrates epigenetic changes and co-activator activity, leading to full induction of gluconeogenesis.

The GCN5-CITED2-PKA signalling module explains why the HAT activity of GCN5 as well as both CITED2 and PKA are required for the gluconeogenic effect of GCN5 overexpression. However, the molecular mechanism by which expression of GCN5(WT) or GCN5(S275A) in the absence of CITED2 overexpression suppresses cAMP- or fasting-induced gluconeogenic gene expression remains to be elucidated. It can be argued that hepatic overexpression of GCN5(WT) alone should promote formation of this signalling module with endogenous CITED2 and PKA, the former of which is upregulated during fasting, and thereby activate gluconeogenesis (Fig. 3c–e). It should be noted, however, that in this setting the abundance of *Gcn5* mRNA in the liver overexpressing GCN5 was 30 times that in the control liver, whereas the amount of CITED2 protein in the liver of fasted mice showed an ∼2.5-fold increase compared with that in refed mice^20^. It is therefore likely that the amount of endogenous CITED2 was insufficient to form the GCN5-CITED2-PKA signalling module with overexpressed GCN5. Given that phosphorylation of GCN5 by PKA is dependent on this module, the excess GCN5 would not be phosphorylated by PKA. As previously described^17^, the suppressive effect of overexpressed GCN5 in the absence of CITED2 overexpression on cAMP- or fasting-induced gluconeogenic gene expression might be mediated by PGC-1α inactivation through acetylation mediated by the nonphosphorylated form of GCN5. However, we found that expression of GCN5(ΔAT) alone also inhibited cAMP-induced gluconeogenic gene expression, suggesting that overexpression of GCN5 suppresses gluconeogenesis through both acetyltransferase-dependent and -independent mechanisms. We have previously shown that coexpression of CITED2 with GCN5(WT) changes the pattern of GCN5 localization in the nucleus from homogeneous to speckled^20^. Overexpressed GCN5 that fails to interact with CITED2 may thus disrupt or affect the localization of the GCN5-CITED2-PKA signalling module independently of its acetyltransferase activity, resulting in suppression of gluconeogenic gene expression.

Depletion of GCN5 or expression of the phosphorylation-defective mutant GCN5(S275A) in the liver of mice with obesity and type 2 diabetes suppressed gluconeogenesis and thereby improved glycemia, suggesting that inhibition of GCN5 expression or phosphorylation at Ser^275^ may ameliorate diabetes. Disruption of the GCN5-CITED2-PKA signalling module (such as that achieved by CITED2 depletion) also suppressed gluconeogenesis and ameliorated diabetes, suggesting that this module is a promising pharmacological target for treatment of obesity and type 2 diabetes.

## Methods

### Mice

All mouse experiments were performed according to procedures approved by the Institutional Animal Care and Use Committee of the National Center for Global Health and Medicine (Tokyo). Lean C57BL/6J male mice (CLEA Japan) as well as *db*/*db* (C57BLKS/J Iar– +*Lepr*^*db*^/+*Lepr*^*db*^) and *db/m* (C57BLKS/J Iar– +*m*^*db*^/+*Lepr*^*db*^) male mice obtained from Institute for Animal Reproduction were studied at 8 weeks of age. Recombinant adenoviruses were injected into the tail vein of mice as described previously^32^ at a dose of 1.0 × 10^9^ or 3.0 × 10^9^ plaque-forming units in a total volume of 0.25 ml for C57BL/6J and *db/db* mice, respectively, and experiments were performed 4 days after adenovirus injection. Blood glucose concentration was measured with the use of a standard glucose sensor (Glutest Ace, Sanwa Kagaku Kenkyusho). For the pyruvate challenge test, mice deprived of food for 16 h were injected intraperitoneally with pyruvate dissolved in saline (2 g kg^−1^) as described previously^33^. For experiments with mice fed a HFD, C57BL/6J mice were maintained on chow containing 30% fat by weight (14% bovine fat, 14% porcine fat, 2% soybean oil; Oriental Yeast) from 4 to 24 weeks of age. For examination of the effects of glucagon, mice were deprived of food for 3 h and then injected intraperitoneally with the hormone at a dose of 100 μg kg^–1^. Liver extracts were prepared 1 h after glucagon treatment. We allocated cages of mice to the experimental groups by random draw. The investigator was not blinded to the group allocation during experiments.

### Plasmids

GCN5, CITED2, PGC-1α, PKACα and PKARIα cDNAs were isolated from C57BL/6J mouse liver and cloned into the mammalian expression vectors pcDNA3 or pcDNA3.1 (Life Technologies). The cDNAs for GCN5(ΔAT) (GYG→AYA/582Y584), GCN5(S275A) and GCN5(S275D) were generated with the use of a KOD-plus Mutagenesis Kit (Toyobo).

### Adenoviruses

Recombinant adenoviruses were constructed with the use of an Adenovirus Dual Expression Kit (Takara Bio). FLAG-GCN5, Myc-GCN5, FLAG-GCN5(ΔAT), FLAG-GCN5(S275A), FLAG-GCN5(S275D), FLAG-CITED2, HA-CITED2, FLAG–PGC-1α, FLAG-FoxO1 and β-galactosidase (control) were expressed under the control of a CAG promoter (cytomegalovirus enhancer, chicken β-actin gene promoter, and rabbit β-globin gene poly(A) signal), whereas shRNAs were expressed under the control of a U6 promoter. The shRNAs for GCN5, CITED2 and PGC-1α were based on the sequences 5′-TGTCAGAGGACGAGATTAA-3′; 5′-TGACGGACTTCGTGTGCA-3′; and 5′-GTATCTGACCACAAACGAT-3′, respectively. A negative control shRNA sequence was obtained from BD Biosciences. Primary hepatocytes or AML12 cells were infected with adenoviruses 1 day after plating. Analyses of gene and protein expression as well as a glucose production assay were performed 2 days after infection.

### Chemicals and antibodies

Glucagon, pCPT-cAMP, insulin, MG132, trichostatin A and acetyl-CoA were obtained from Sigma; H89 and nicotinamide were from Enzo Life Sciences and Nacalai Tesque, respectively; recombinant histone H3.1 was from New England Biolabs; and recombinant human CITED2 was from Abcam. Antibodies to Myc (#2276, 1:1,000 dilution; #2272, 1:1,000), to histone H3 (#4499, 1:1,000), to H3K9ac (#9649, 1:1,000), to acetylated lysine (#9441, 1:1,000), to Ser/Thr–phosphorylated PKA substrates (#9621, 1:1,000), to CREB (#9197, 1:1,000), to Ser^133^-phosphorylated CREB (#9198, 1:1,000), to IP3R1 (#8568, 1:1,000), to Ser^1756^-phosphorylated IP3R (#8548, 1:1,000), to VASP (#3132, 1:1,000), to Ser^157^-phosphorylated VASP (#3111, 1:1,000), to PKACα (#5842, 1:1,000) and to PKARIα (#5675, 1:1,000) were obtained from Cell Signaling. Antibodies to GCN5 (sc-20698, 1:500; sc-365321, 1:500), to PCAF (sc-13124, 1:500), to CBP (sc-51517, 1:500; sc-369), to HNF-4α (sc-8987), to PGC-1α (sc-67285, 1:500) and to histone H1 (sc-10806, 1:400) were obtained from Santa Cruz Biotechnology. Antibodies to CITED2 (ab108345, 1:1,000), to H3K9ac (ab4441, 1:10,000) and to H3K27ac (ab4729) were from Abcam; those to p300 (05-257, 1:1,000), to CRTC2 (ST-1099, 1:2,000), and to H3K4me3 (05-745R) were from Millipore; those to FLAG (F1804, 1:1,000) and to β-actin (A5441, 1:10,000) were from Sigma; those to HA (11867423001, 1:2,000) and to His_6_ (04905318001, 1:500) were from Roche; and those to V5 (R960, 1:2,000), to FLAG (KO602, 1:2,000), to DsRed (for mCherry; 632496, 1:1,000), and to T7 (69522, 1:1,000) were from Life Technologies, Transgenic, Clontech, and Novagen, respectively. Rabbit antibodies to Ser^275^-phosphorylated GCN5 (1:400) were generated in response to a phosphopeptide containing amino acids 271 to 280 of mouse GCN5.

### Cell culture

Primary hepatocytes were isolated from 8- to 12-week-old male C57BL/6J mice fed a normal chow diet as described previously^32^. In brief, mice were anaesthetised by intraperitoneal injection of medetomidine (0.75 mg kg^−1^), midazolam (4 mg kg^−1^) and butorphanol (5 mg kg^−1^), and the liver was perfused at a rate of 4.5 ml min^−1^ first for 3–5 min with oxygenated Hanks' balanced salt solution containing 10 mM Hepes-NaOH (pH 7.4) and then for 18 min with the same solution containing collagenase type I (30–32 mg per 100 ml, Worthington) and Protease Inhibitor Cocktail Complete–EDTA Free (one tablet per 50 ml, Roche). The hepatocytes were harvested and purified by density gradient centrifugation with Percoll (Sigma), and their viability was assessed on the basis of trypan blue exclusion. We studied only hepatocyte preparations with a viability of >90%. The cells were plated on type I collagen–coated six-well plates (1.0 × 10^6^ cells per well) in Medium 199 (Life Technologies) supplemented with 5% fetal bovine serum, and were incubated overnight in serum-free Medium 199 (Life Technologies) before the addition of pCPT-cAMP (100 μM) (ref. 34). AML12 and HEK293 cells were obtained from American Type Culture Collection. AML12 cells were cultured in a 1:1 (v/v) mixture of Dulbecco's modified Eagle's medium (DMEM) and Ham's F12 medium that was supplemented with insulin (5 μg ml^–1^), transferrin (5 μg ml^–1^), selenium (5 ng ml^–1^), dexamethasone (40 ng ml^–1^), and 10% fetal bovine serum, whereas HEK293 cells were cultured in DMEM supplemented with 10% fetal bovine serum. Primary hepatocytes and AML12 cells were tested for gluconeogenic gene induction by pCPT-cAMP. Where indicated, primary hepatocytes or AML12 cells were exposed to 20 μM H89 for 30 min before incubation in the additional presence of 100 μM pCPT-cAMP for the indicated times. The cell lines were regularly tested for mycoplasma contamination.

### Gene expression analysis

Total RNA was isolated from cells or pulverized liver with the use of an RNeasy Mini Kit and RNase-Free DNase Set (Qiagen). For quantitative RT-PCR analysis, cDNA was synthesized from the total RNA with the use of random primers and a High Capacity cDNA Reverse Transcription Kit (Life Technologies) and PCR was then performed in triplicate with the use of a StepOnePlus Real-Time PCR System and Power SYBR Green PCR Master Mix (Life Technologies). Relative mRNA abundance was calculated by the standard curve method and was normalized by the corresponding amount of 18S rRNA. Primer sequences are listed in Supplementary Table 1.

### Protein interaction analysis

Protein-protein interactions in cells were examined with co-IP assays. Epitope-tagged proteins were expressed in AML12 cells or HEK293 cells by transfection with the use of a Nucleofector Kit V (Amaxa) or by adenoviral transduction. The cells were then lysed in a lysis buffer containing 20 mM Tris-HCl (pH 7.5), 150 mM NaCl, 0.5% Nonidet P-40, 2 mM EDTA, 10% glycerol, 10 mM nicotinamide, 1 μM trichostatin A, 10 μM MG132, and protease and phosphatase inhibitors (Roche), and the lysates were subjected to IP with the indicated antibodies and either protein G-Sepharose (GE Healthcare) or Dynabeads Protein G (VERITAS). The immunoprecipitates were fractionated by SDS–polyacrylamide gel electrophoresis (PAGE) and subjected to immunoblot analysis with the indicated antibodies. Uncropped images of representative immunoblots are shown in Supplementary Fig. 8.

### Preparation of nuclear extracts from mouse liver

The liver was removed, rinsed in ice-cold phosphate-buffered saline (PBS), suspended in 5 ml of buffer A (10 mM Hepes-NaOH (pH 7.9), 25 mM KCl, 1 mM EDTA, 2 M sucrose, 10% glycerol, 10 mM nicotinamide, 1 μM trichostatin A, 10 μM MG132 and protease and phosphatase inhibitors (Roche)), and homogenized briefly with a Polytron disruptor. The homogenate (15 ml) was passed five times through a 20 G needle and then layered on top of 20 ml of buffer A in a centrifuge tube and centrifuged at 100,000*g* for 1 h at 4 °C. The resulting nuclear pellet was washed with 300 μl of a solution containing 10 mM Hepes-NaOH (pH 7.9), 100 mM KCl, 2 mM MgCl_2_, 1 mM EDTA and 10% glycerol and was then digested with Enzymatical Shearing Cocktail in 200 μl of Complete Digestion Buffer (Nuclear Complex Co-IP Kit, Active Motif). The reaction was terminated by the addition of 4 μl of 0.5 M EDTA, and the mixture was then centrifuged at 14,000*g* for 10 min at 4 °C. The resulting supernatant was collected as the nuclear extract for co-IP experiments.

### Glucose production assay

Primary hepatocytes were cultured in serum-free Medium 199 in the absence or presence of 100 μM pCPT-cAMP for 16 h as previously described^34^. They were then incubated for 6 h in glucose- and phenol red-free DMEM (pH 7.4) supplemented with sodium lactate and pyruvate before measurement of glucose released into the medium with the use of a colorimetric assay (Wako)^7^. Data are presented as arbitrary units (AU).

### *In vitro* assay of HAT activity

Nuclear extracts of AML12 cells expressing epitope-tagged GCN5 were subjected to IP with monoclonal antibodies to Myc (9B11, #2276) or to FLAG (2H8, KO602) together with protein G-Sepharose. The immunoprecipitates were mixed with 30 μl of acetylation buffer (40 mM Tris-HCl (pH 8.0), 0.1 M NaCl, 10% glycerol, 0.1 mM EDTA, 1 mM dithiothreitol, 1 mM phenylmethylsulfonyl fluoride (PMSF), 1 μM trichostatin A, 0.1 mM acetyl-CoA) containing 1 μg of recombinant histone H3.1 with or without 3 μg of a recombinant His_6_-tagged NH_2_-terminal fragment (residues 1–400) of mouse PGC-1α produced in bacteria. The reaction mixtures were incubated for 90 min at 30 °C, the reaction was terminated by the addition of 40 μl of 2 × SDS sample buffer and heating at 100 °C, and a portion (10 μl) of each mixture was subjected to SDS–PAGE on a 15% gel followed by immunoblot analysis.

### *In vitro* kinase assay

FLAG immunoprecipitates prepared from HEK293 cells expressing FLAG-GCN5(WT) or FLAG-GCN5(S275A) were incubated for 30 min at 30 °C with or without 500 ng of glutathione S-transferase (GST)–tagged PKACα (R&D Systems) and 50 μM H89 in 30 μl of kinase buffer (25 mM Tris-HCl (pH 7.5), 10 mM MgCl_2_, 5 mM β-glycerophosphate, 0.2 mM EGTA, 2 mM dithiothreitol, 100 μM ATP and 1 μCi of [γ-^32^P]ATP). The reaction mixtures were subjected to SDS–PAGE, and phosphorylated substrates were detected by autoradiography and analysis with a BAS2500 system (Fujifilm).

### ChIP-qPCR analysis

ChIP was performed 2 or 4 days after adenoviral infection of primary hepatocytes or mice, respectively. Primary hepatocytes or minced liver tissue from mice were subjected to crosslinking by incubation with 1% formaldehyde for 10 min at room temperature. The reaction was quenched by the addition of glycine to a final concentration of 125 mM, after which the liver tissue was subjected to Dounce homogenization in cell lysis buffer (5 mM Pipes-NaOH (pH 8.0), 85 mM KCl, 0.5% Nonidet P-40, protease inhibitor cocktail (Roche)) for preparation of a nuclear extract. The hepatocytes were washed twice with PBS, suspended in PBS containing 0.5% IGEPAL CA-630 (Sigma-Aldrich), protease inhibitor cocktail, and 1 mM PMSF, and isolated by centrifugation. Chromatin fragmentation was performed by ultrasonic treatment (Bioruptor UCW-310 sonicator) of the cells or liver nuclear extract in 250 μl of SDS lysis buffer (50 mM Tris-HCl (pH 8.0), 10 mM EDTA, 1% SDS, protease inhibitor cocktail, 1 mM PMSF) to yield an average DNA size of 500 bp. The samples were then cleared by centrifugation (13,000*g* for 15 min at 4 °C), and the resulting supernatants (110 μl) were mixed with 990 μl of dilution buffer (5.56 mM Tris-HCl (pH 7.6), 167 mM NaCl, 0.11% sodium deoxycholate, 1.11% Triton X-100, protease inhibitor cocktail). The diluted samples were incubated with 50 μl of Dynabeads Protein G for 1 h at 4 °C with rotation, the beads were discarded, and 10% of the supernatant was saved for analysis as ChIP input. The protein of interest was immunoprecipitated by incubation of the remaining supernatant overnight at 4 °C with rotation in the presence of 50 μl of Dynabeads Protein G coated with specific antibodies. The beads were collected with a magnet, resuspended in 1 ml of low-salt wash buffer (10 mM Tris-HCl (pH 7.6), 1 mM EDTA, 150 mM NaCl, 0.1% SDS, 0.1% sodium deoxycholate, 1% Triton X-100, protease inhibitor cocktail), and washed twice each with low-salt wash buffer, high-salt wash buffer (same as low-salt wash buffer but containing 500 mM NaCl), LiCl wash buffer (10 mM Tris-HCl (pH 7.6), 1 mM EDTA, 250 mM LiCl, 0.5% IGEPAL CA-630, 0.5% sodium deoxycholate, protease inhibitor cocktail) and TE (Tris-EDTA) buffer containing protease inhibitor cocktail. Crosslinks were reversed by incubation of the immunoprecipitates for 6 h at 65 °C in 100 μl of TE buffer plus 3 μl of 10% SDS and 5 μl of proteinase K (20 mg ml^–1^). Genomic DNA was isolated with the use of a MinElute PCR Purification Kit (Qiagen) and was quantified by PCR analysis relative to input DNA. Primer sequences are listed in Supplementary Table 1.

### Proximity ligation assay

The PLA^26^ was performed to visualize protein interaction *in situ* with the use of Duolink *In Situ* PLA reagents (Olink Bioscience).

### RNA interference with siRNA

AML12 cells were transfected with control or mouse PKACα SMARTpool small interfering RNAs (Dharmacon) with the use of a Nucleofector Kit V (Amaxa). Analysis of protein interaction and *in vitro* assay of HAT activity were performed 2 days after transfection.

### *In silico* analysis of putative PKA phosphorylation sites of GCN5

Prediction of phosphorylation motifs (R–X–X–S/T) of GCN5 conserved between rodents (mouse and rat) and human was performed with the use of GPS2.1 software (Group-based Prediction System, version 2.1)^35^.

### Statistical analysis

Quantitative data are presented as means±s.e.m. No statistical method was used to predetermine sample size, which was based on preliminary data and previous publications. Each experiment was performed at least three times. Animals were excluded from experiments if they showed any sign of sickness. Results were evaluated with two-tailed Student's *t*-test or one-way or two-way analysis of variance, as appropriate, with the use of GraphPad Prism software. Welch's *t*-test was applied when variance among groups was found to differ. Significant differences revealed by analysis of variance were assessed with Bonferroni's test. A *P* value of <0.05 was considered statistically significant.

### Data availability

The data that support the findings of this study are available from the corresponding author on request.

## Additional information

**How to cite this article:** Sakai, M. *et al*. The GCN5-CITED2-PKA signalling module controls hepatic glucose metabolism through a cAMP-induced substrate switch. *Nat. Commun.* **7,** 13147 doi: 10.1038/ncomms13147 (2016).

**Publisher's note**: Springer Nature remains neutral with regard to jurisdictional claims in published maps and institutional affiliations.

## Supplementary Material

Supplementary Information Supplementary Figures 1-8 and Supplementary Table 1.
