# Supplementary material for: The GCN5-CITED2-PKA signalling module controls hepatic glucose metabolism through a cAMP-induced substrate switch
Source: Nat Commun. 2016 Nov 22;7:13147. doi: 10.1038/ncomms13147 (PMC5121418; doi:10.1038/ncomms13147)
Supplement: Supplementary Information — Supplementary Figures 1-8 and Supplementary Table 1. [file ncomms13147-s1.pdf]

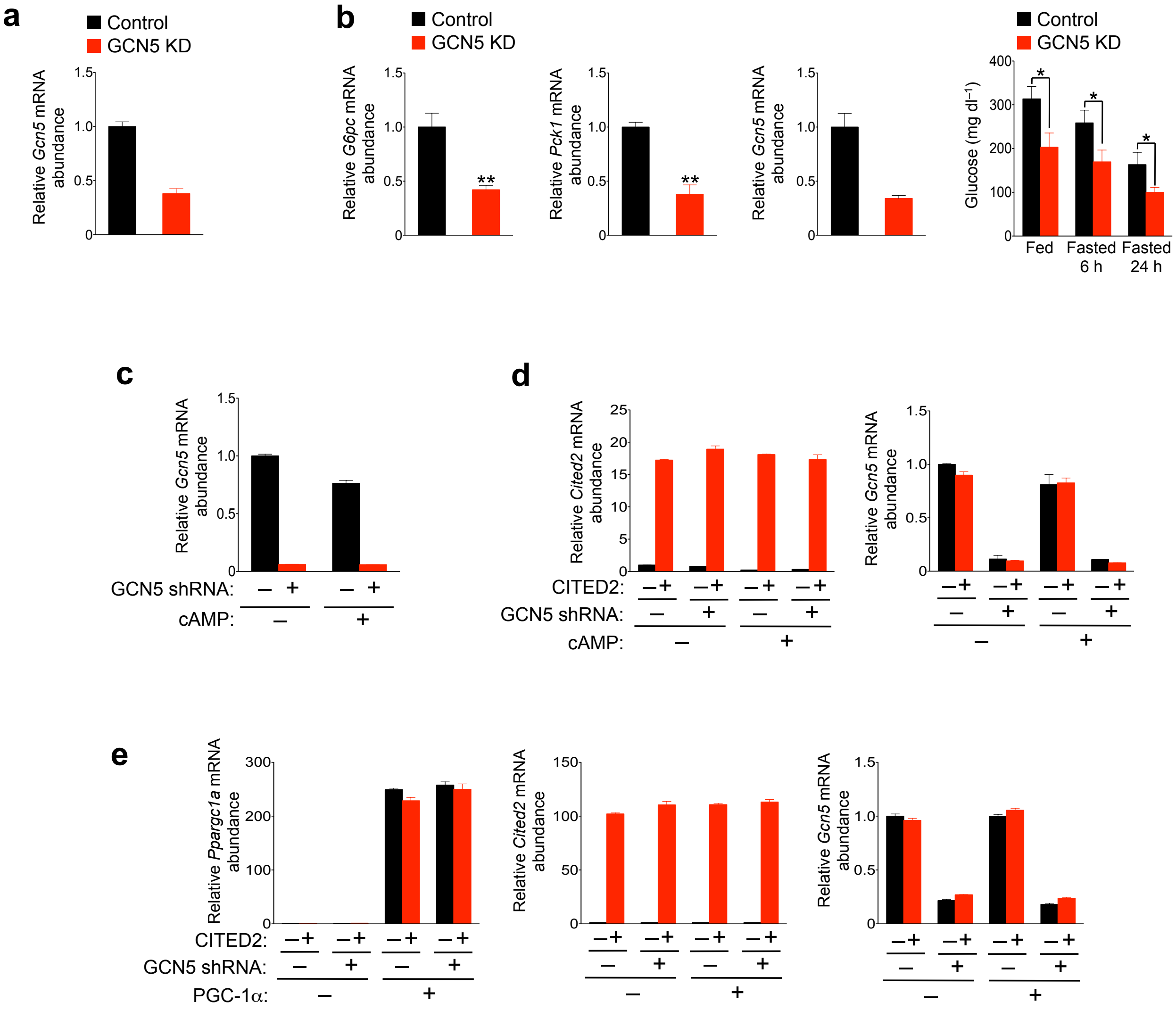

Supplementary Figure 1. (**a**) Quantitative RT-PCR analysis of *Gcn5* mRNA in the liver of C57BL/6J mice injected with an adenovirus for GCN5 shRNA and deprived of food for 24 h. See **Figure 2a**. (**b**) Effects of shRNA-mediated knockdown of GCN5 in the liver of *db/db* mice on hepatic expression of *G6pc*, *Pck1*, and *Gcn5* under the fasted (24 h) condition as well as on plasma glucose concentration under fed or fasted (6 or 24 h) conditions. Data are means ± s.e.m. (control (n = 8), GCN5 KD (n = 9)). *P < 0.05, **P < 0.01 versus control or as indicated (unpaired Student's t test). (**c**) Quantitative RT-PCR analysis of *Gcn5* mRNA in primary hepatocytes depleted of GCN5 and exposed (or not) to pCPT-cAMP. See **Figure 2d**. (**d**) Quantitative RT-PCR analysis of *Cited2* and *Gcn5* expression in primary hepatocytes depleted (or not) of GCN5 in the absence or presence of CITED2 overexpression and with or without exposure to pCPT-cAMP for 6 h. See **Figure 2f**. (**e**) Quantitative RT-PCR analysis of *Ppargc1a* (PGC-1α), *Cited2*, and *Gcn5* mRNAs in primary hepatocytes depleted (or not) of GCN5 with or without overexpression of PGC-1α or CITED2. See **Figure 2g**.

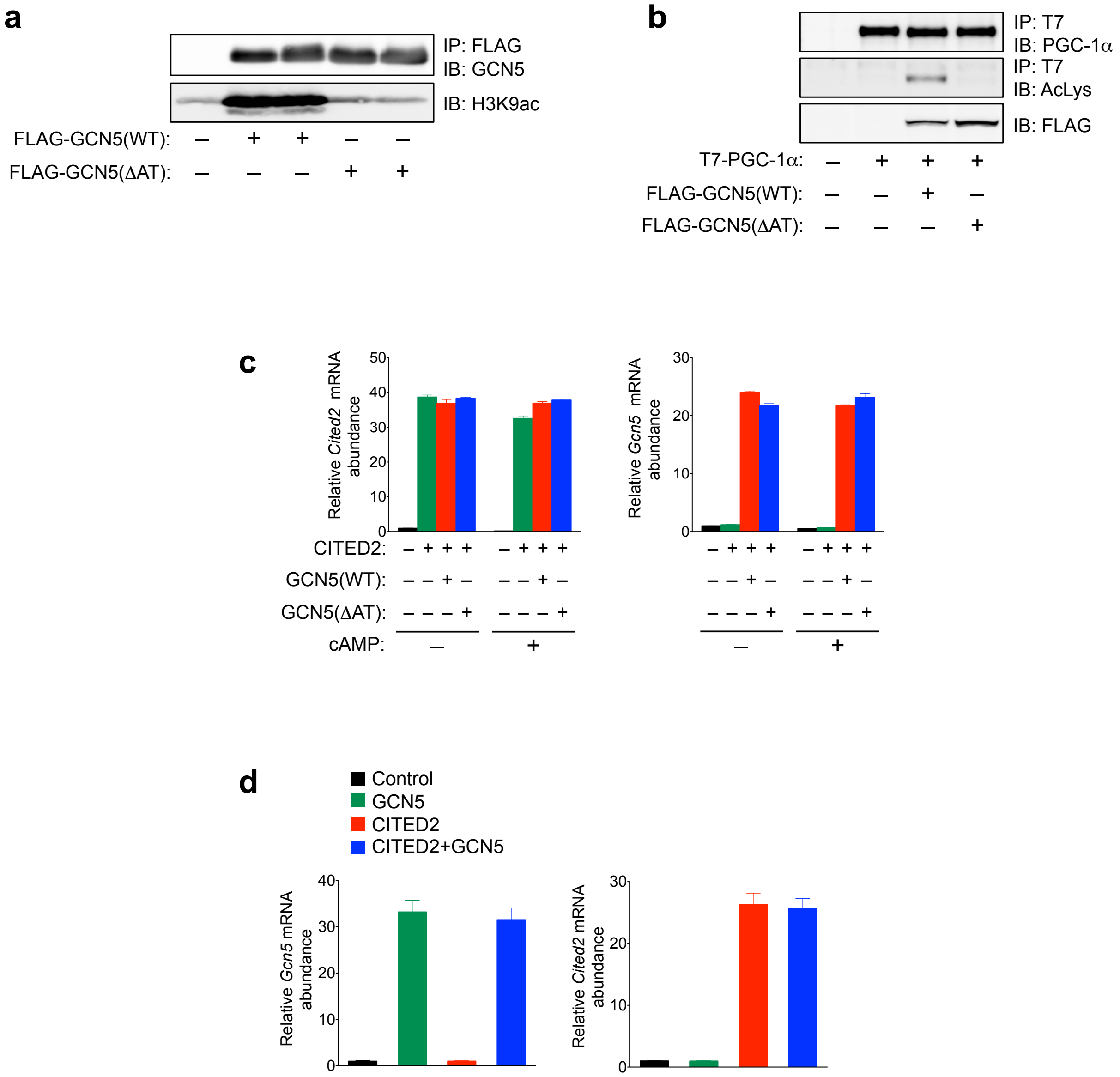

Supplementary Figure 2. (**a**) Lack of HAT activity of immunoprecipitated FLAG-GCN5(ΔAT) as assayed *in vitro* with histone H3 as substrate. (**b**) Acetylation of T7-tagged PGC-1α in HEK293 cells expressing FLAG-GCN5(ΔAT) or FLAG-GCN5(WT). Data in **a** and **b** are representative of at least three independent experiments. (**c**) Quantitative RT-PCR analysis of *Cited2* and *Gcn5* mRNAs in primary hepatocytes expressing GCN5(WT) or GCN5(ΔAT) with or without CITED2 overexpression and with or without exposure to pCPT-cAMP for 6 h. See **Figure 3b**. (**d**) Quantitative RT-PCR analysis of *Gcn5* and *Cited2* mRNAs in the liver of C57BL/6J mice overexpressing GCN5, CITED2, or both proteins and deprived of food for 24 h. See **Figure 3d**.

Supplementary Figure 3

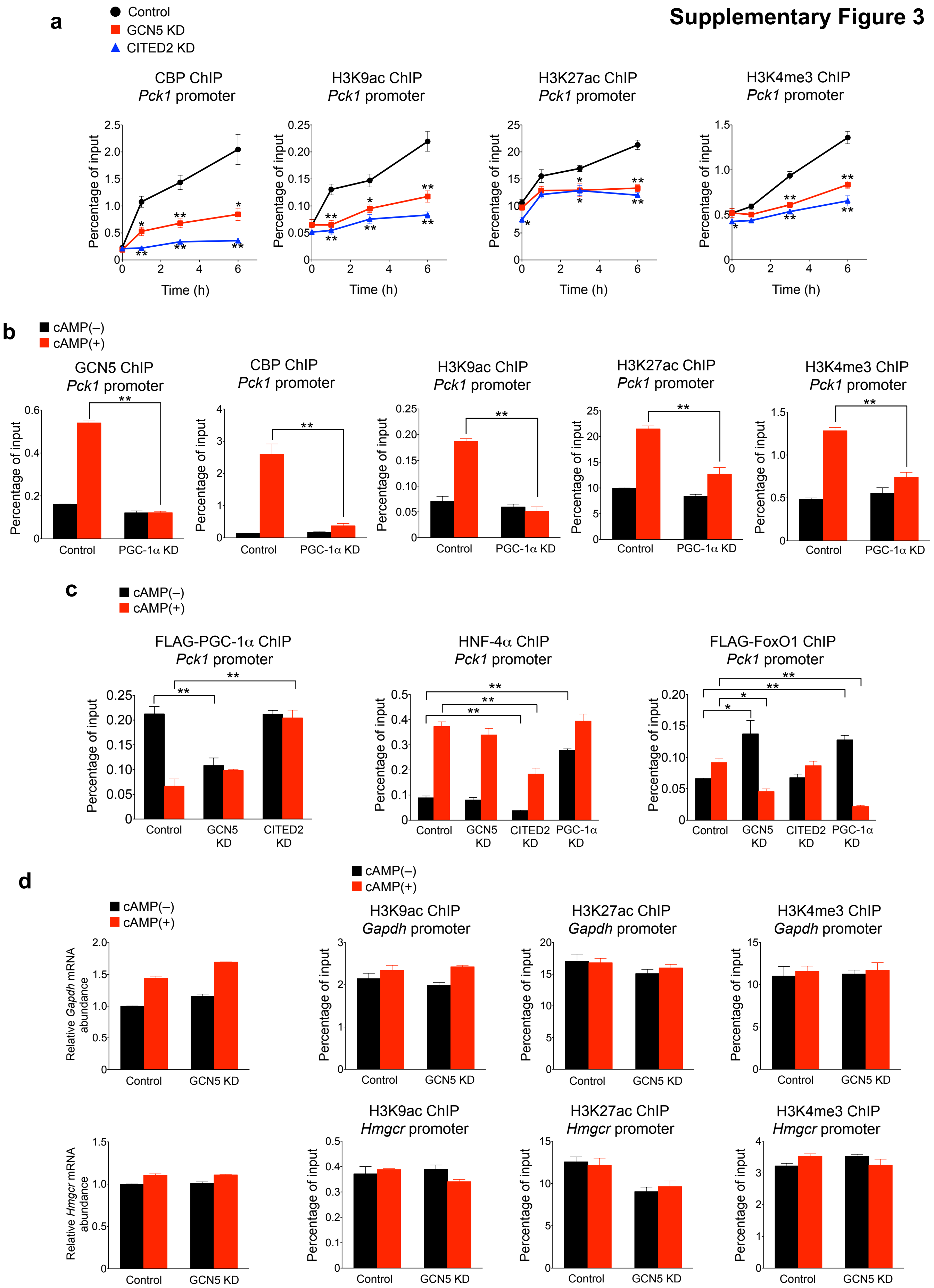

e

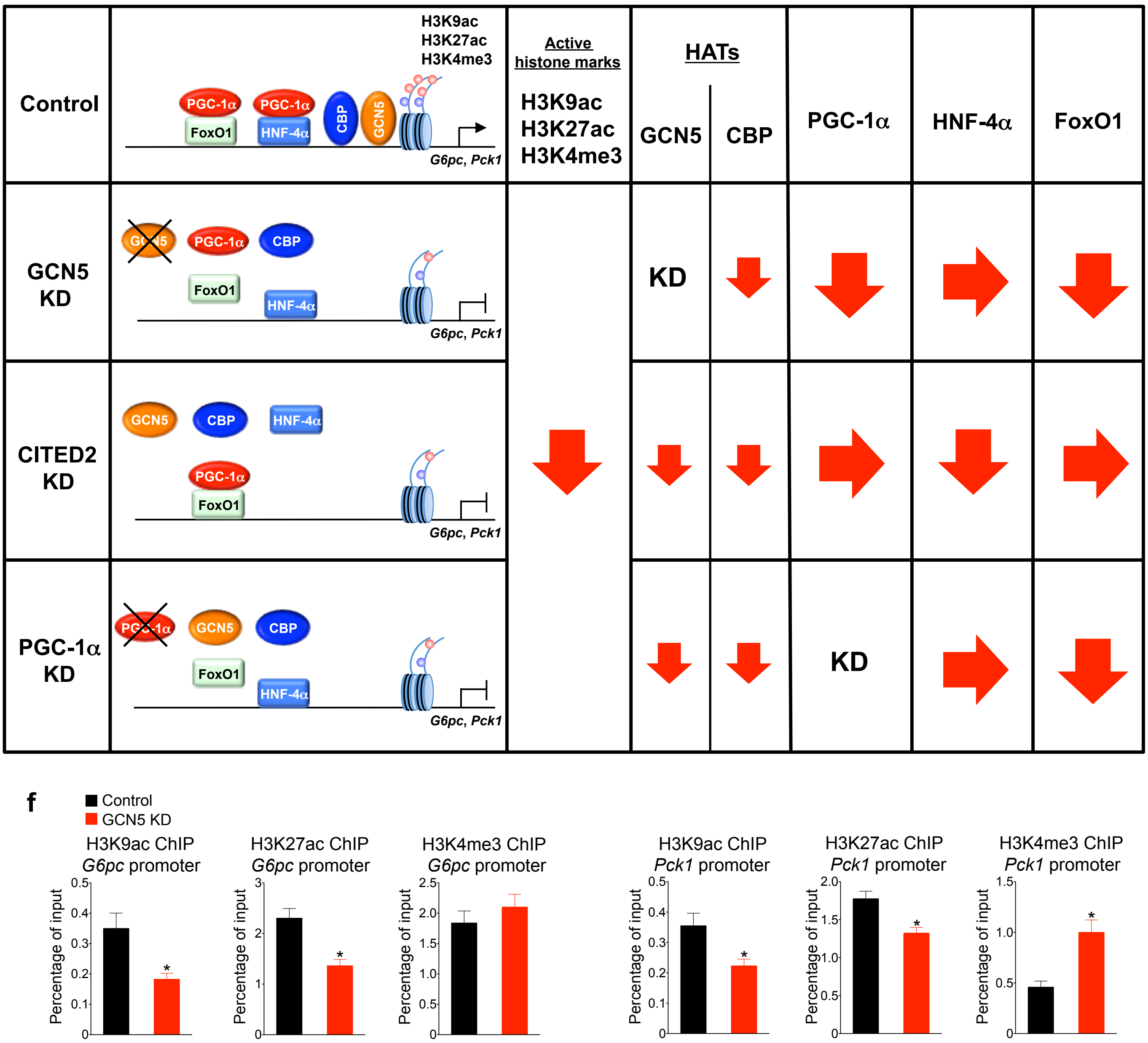

Supplementary Figure 3. (**a**) ChIP-qPCR analysis of the occupancy of the *Pck1* promoter with CBP or epigenomic marks in primary hepatocytes depleted of GCN5 or CITED2 and exposed to pCPT-cAMP for the indicated times. (**b**) ChIP-qPCR analysis of the occupancy of the *Pck1* promoter with GCN5, CBP, or epigenomic marks in primary hepatocytes depleted of PGC-1α and exposed to pCPT-cAMP for 6 h. (**c**) ChIP-qPCR analysis of the occupancy of the *Pck1* promoter with FLAG–PGC-1α, HNF-4α, or FLAG-FoxO1 in primary hepatocytes depleted of GCN5, CITED2, or PGC-1α and exposed to pCPT-cAMP for 6 h. (**d**) Quantitative RT-PCR analysis of *Gapdh* and *Hmgcr* mRNAs (left) as well as ChIP-qPCR analysis of the occupancy of the *Gapdh* and *Hmgcr* promoters with epigenomic marks (right) in primary hepatocytes depleted of GCN5 and exposed to pCPT-cAMP for 6 h. (**e**) Summary of the results of ChIP-qPCR assays for gluconeogenic gene promoters in hepatocytes depleted of GCN5, CITED2, or PGC-1α and exposed to pCPT-cAMP for 6 h. KD, knockdown. (**f**) ChIP-qPCR analysis of the occupancy of the *G6pc* and *Pck1* promoters with epigenomic marks in the liver of C57BL/6J mice injected with an adenovirus for GCN5 shRNA and deprived of food for 16 h. All data are means ± s.e.m. (n = 3). *P < 0.05, **P < 0.01 versus control or as indicated (ANOVA with Bonferroni's *post hoc* test (**a–c**) or unpaired Student's t test (**f**)). Adenoviral vectors encoding GCN5, CITED2, or PGC-1α shRNAs, FLAG-PGC-1α, or FLAG-FoxO1 were used for these experiments.

Supplementary Figure 4

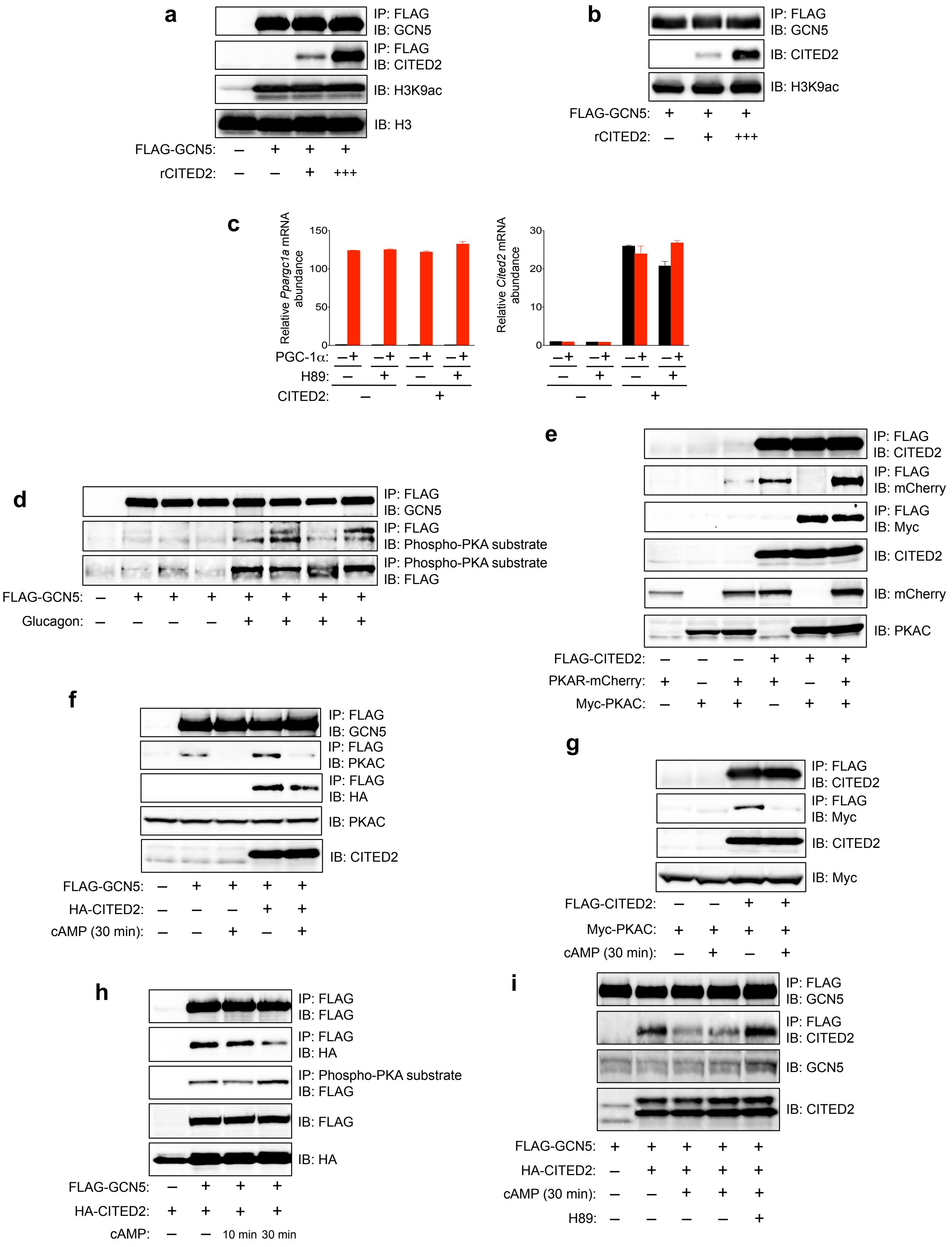

Supplementary Figure 4. (**a, b**) Binding of recombinant CITED2 to FLAG-GCN5 *in vitro* and its lack of effect on HAT activity. AML12 cells expressing FLAG-GCN5 were subjected to immunoprecipitation with antibodies to FLAG in the absence or presence of recombinant CITED2 (rCITED2), and the resulting immunoprecipitates were assayed for HAT activity *in vitro* with recombinant histone H3 as substrate (**a**). FLAG-GCN5 immunoprecipitates were also assayed for HAT activity *in vitro* with or without recombinant CITED2 in the reaction mixture (**b**). (**c**) Quantitative RT-PCR analysis of *Ppargc1a* and *Cited2* mRNAs in primary hepatocytes infected with adenoviruses encoding PGC-1α or CITED2 and incubated in the absence or presence of H89 (20 μM, 6 h). See **Figure 6a**. (**d**) Glucagon-induced phosphorylation of FLAG-GCN5 in mouse liver. C57BL/6J mice expressing FLAG-GCN5 in the liver were deprived of food and then injected intraperitoneally with glucagon. Liver extracts were subjected to immunoprecipitation with antibodies to FLAG or to phosphorylated PKA substrates, and the resulting immunoprecipitates were subjected to immunoblot analysis with the indicated antibodies. (**e**) Immunoprecipitation and immunoblot analysis of the interaction of FLAG-CITED2 with mCherry-tagged PKAR or Myc-PKAC in AML12 cells. (**f**) Immunoblot analysis of FLAG-GCN5 immunoprecipitates from AML12 cells showing the effect of pCPT-cAMP treatment on interaction of FLAG-GCN5 with endogenous PKAC and HA-CITED2. (**g**) Effect of pCPT-cAMP treatment on the interaction of FLAG-CITED2 with Myc-PKAC in AML12 cells. (**h**) Immunoprecipitation and immunoblot analysis of the reciprocal effects of pCPT-cAMP treatment on the interaction of FLAG-GCN5 with HA-CITED2 as well as on PKA-dependent phosphorylation of FLAG-GCN5 in AML12 cells. (**i**) Effects of pCPT-cAMP and H89 on the interaction of FLAG-GCN5 with HA-CITED2 in AML12 cells. Myc-PKAC and PKAR-mCherry plasmids were introduced into cells by transfection, whereas adenoviral vectors were used to introduce other epitope-tagged (or not) proteins as indicated in these experiments. All immunoblot data are representative of at least three independent experiments.

Supplementary Figure 5

a

Putative PKA-specific phosphorylation sites

Position	Peptide	Score
275	P A Q F R Q R S Q S E D V A T	4.003
777	T M T E R L R S R Y Y V T R K	2.441
482	A N A A R D E T A R L E E R R	1.865
812	S E Y C R C A S A L E K F F Y	1.270
308	D S L P R Y E T T H V F G R S	1.168

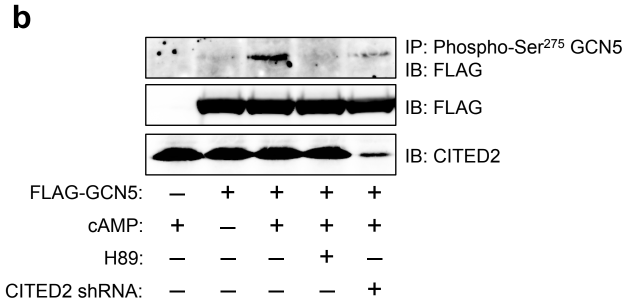

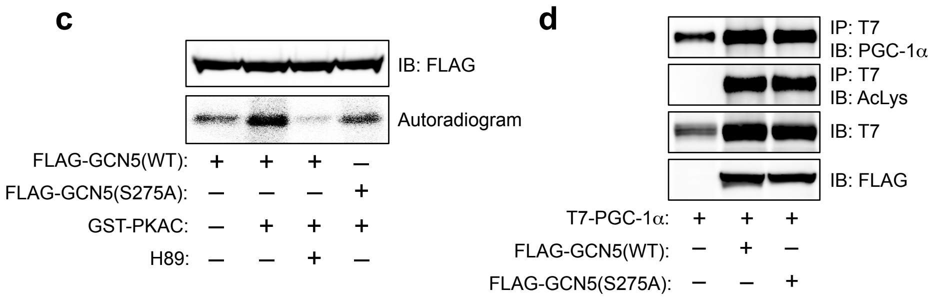

Supplementary Figure 5. (**a**) Putative PKA phosphorylation sites in mouse GCN5 conserved between human and rodents. (**b**) Primary mouse hepatocytes expressing FLAG-GCN5 or CITED2 shRNA as indicated were exposed (or not) to pCPT-cAMP (30 min) with or without H89 and then subjected to immunoprecipitation with antibodies to Ser^{275}-phosphorylated GCN5 followed by immunoblot analysis with antibodies to FLAG. (**c**) *In vitro* kinase assay performed in the absence or presence of H89 (50 μM) with recombinant GST-tagged PKACα and FLAG immunoprecipitates prepared from HEK293 cells expressing FLAG-tagged GCN5(WT) or GCN5(S275A) as substrate. (**d**) Effect of the S275A mutation of FLAG-GCN5 on acetyltransferase activity toward T7-tagged PGC-1α in HEK293 cells. All data are representative of at least three independent experiments. Adenoviral vectors for the indicated tagged protein or shRNA constructs were used for these experiments.

Supplementary Figure 6

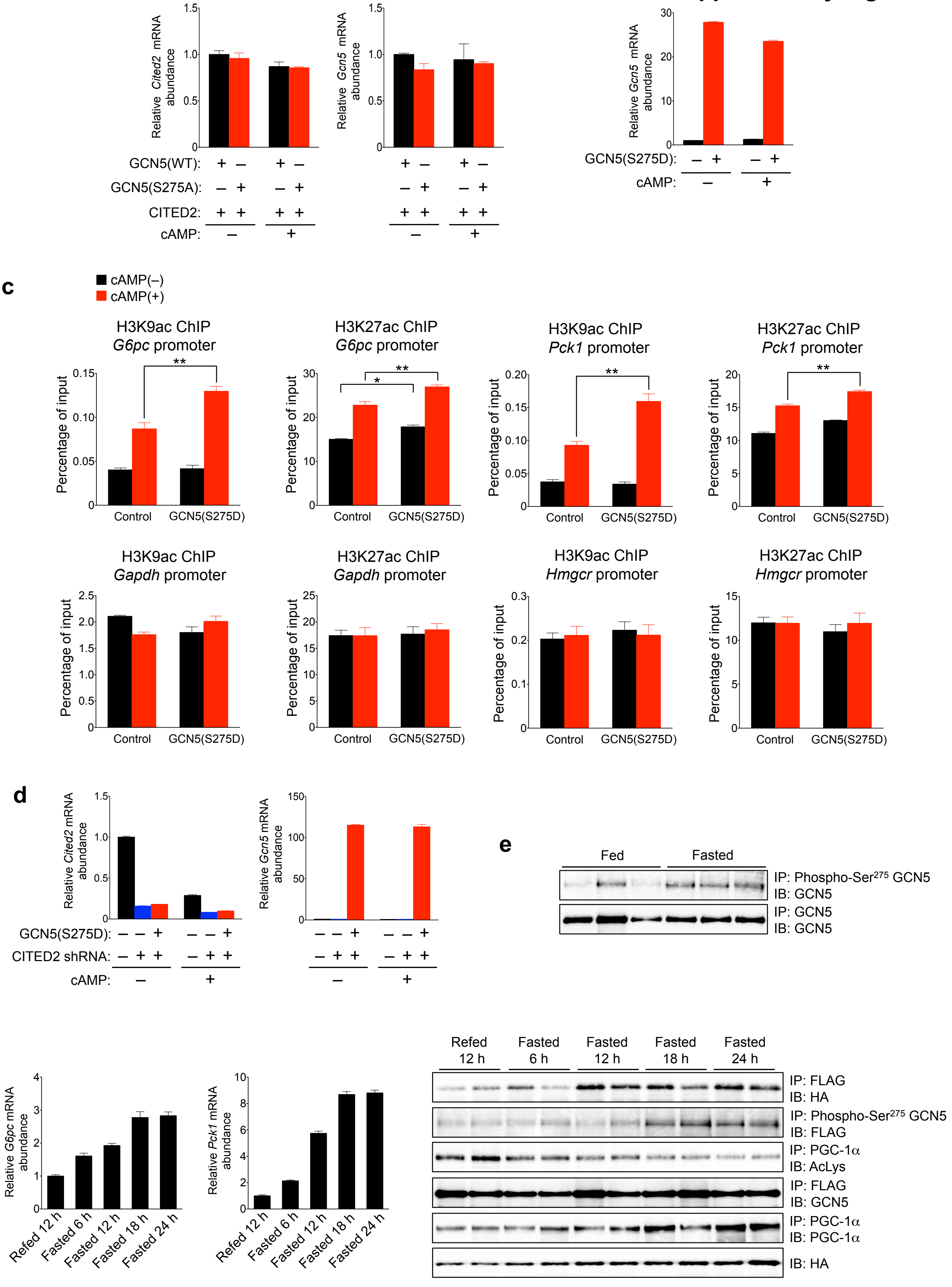

Supplementary Figure 6. (**a**) Quantitative RT-PCR analysis of *Cited2* and *Gcn5* expression in primary mouse hepatocytes infected with adenoviruses encoding CITED2 and either WT or S275A mutant forms of GCN5 and exposed (or not) to pCPT-cAMP for 6 h. See **Figure 8a**. (**b**) Quantitative RT-PCR analysis of *Gcn5* expression in primary mouse hepatocytes infected with an adenovirus encoding GCN5(S275D) and incubated in the absence or presence of pCPT-cAMP for 6 h. See **Figure 8b**. (**c**) ChIP-qPCR analysis of the occupancy of the *G6pc*, *Pck1*, *Gapdh*, and *Hmgcr* promoters with epigenomic marks in primary hepatocytes expressing GCN5(S275D) and exposed to pCPT-cAMP for 6 h. (**d**) Quantitative RT-PCR analysis of *G6pc*, *Pck1*, *Cited2* and *Gcn5* expression in primary mouse hepatocytes infected with adenoviruses encoding CITED2 shRNA or GCN5(S275D) and exposed (or not) to pCPT-cAMP for 6 h. See **Figure 8d**. (**e**) Immunoprecipitation and immunoblot analysis of GCN5 phosphorylated at Ser^{275} in mouse liver. Liver extracts from C57BL/6J mice that had been allowed to feed freely or deprived of food for 16 h were subjected to immunoprecipitation with antibodies to Ser^{275}-phosphorylated GCN5 followed by immunoblot analysis with antibodies to GCN5. (**f**) Time course analysis of gluconeogenic gene expression, GCN5-CITED2 interaction (as assessed by co-immunoprecipitation of HA-CITED2 with FLAG-GCN5), phosphorylation of GCN5 at Ser^{275}, and acetylation of PGC-1α in mouse liver during fasting after refeeding for 12 h. C57BL/6J mice expressing FLAG-GCN5 and HA-CITED2 in the liver were allowed to refeed for 12 h after an overnight fast (Refed 12 h) and were then deprived of food for the indicated times. Data in **c** are means ± s.e.m. (n = 3). $^*P < 0.05$, $^{**}P < 0.01$ (ANOVA with Bonferroni's *post hoc* test). Data in **e** and **f** are representative of at least three independent experiments. Adenoviral vectors for the indicated protein or shRNA constructs were used for these experiments.

Supplementary Figure 7

a

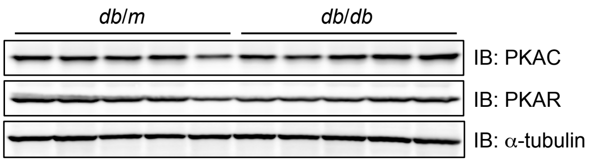

b

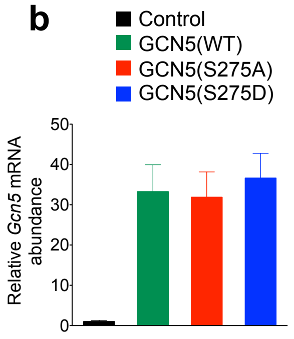

Supplementary Figure 7. (**a**) Immunoblot analysis of PKAC and PKAR in the liver of *db/db* or *db/m* mice (n = 5). (**b**) RT-qPCR analysis of hepatic *Gcn5* mRNA in *db/db* mice expressing GCN5(WT), GCN5(S275A), or GCN5(S275D) in the liver and deprived of food for 24 h. See Figure 9c.

Supplementary Figure 8.
Uncropped images of original scans of representative immunoblots.

Fig. 1a

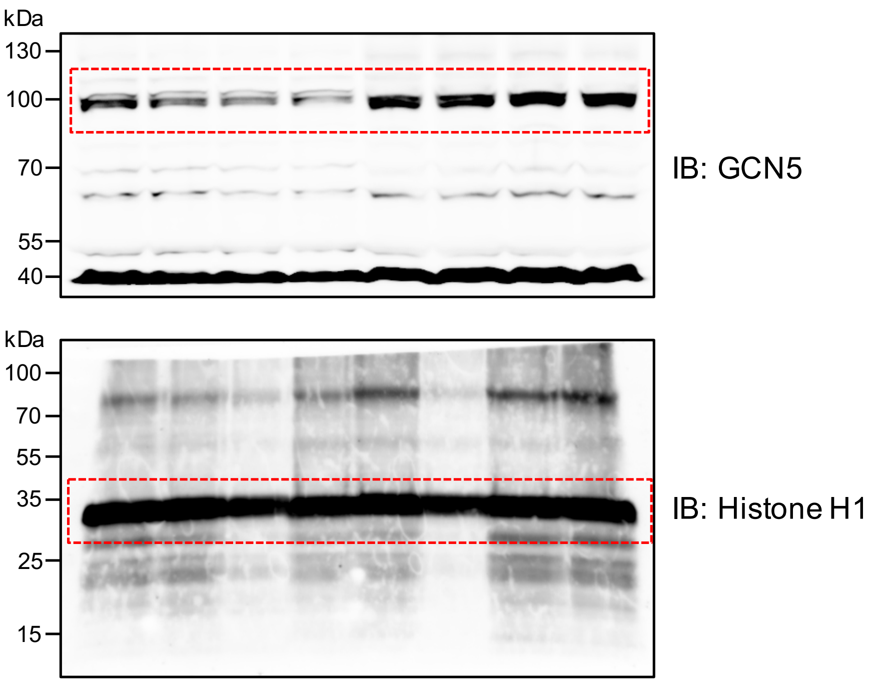

Fig. 1b

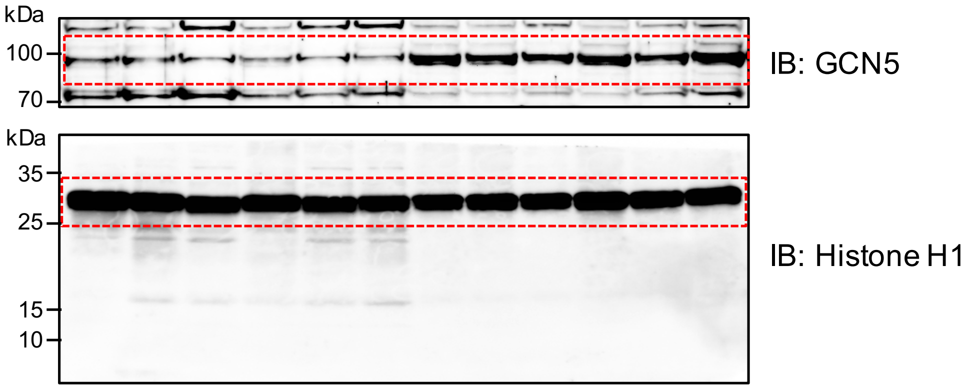

Fig. 1c

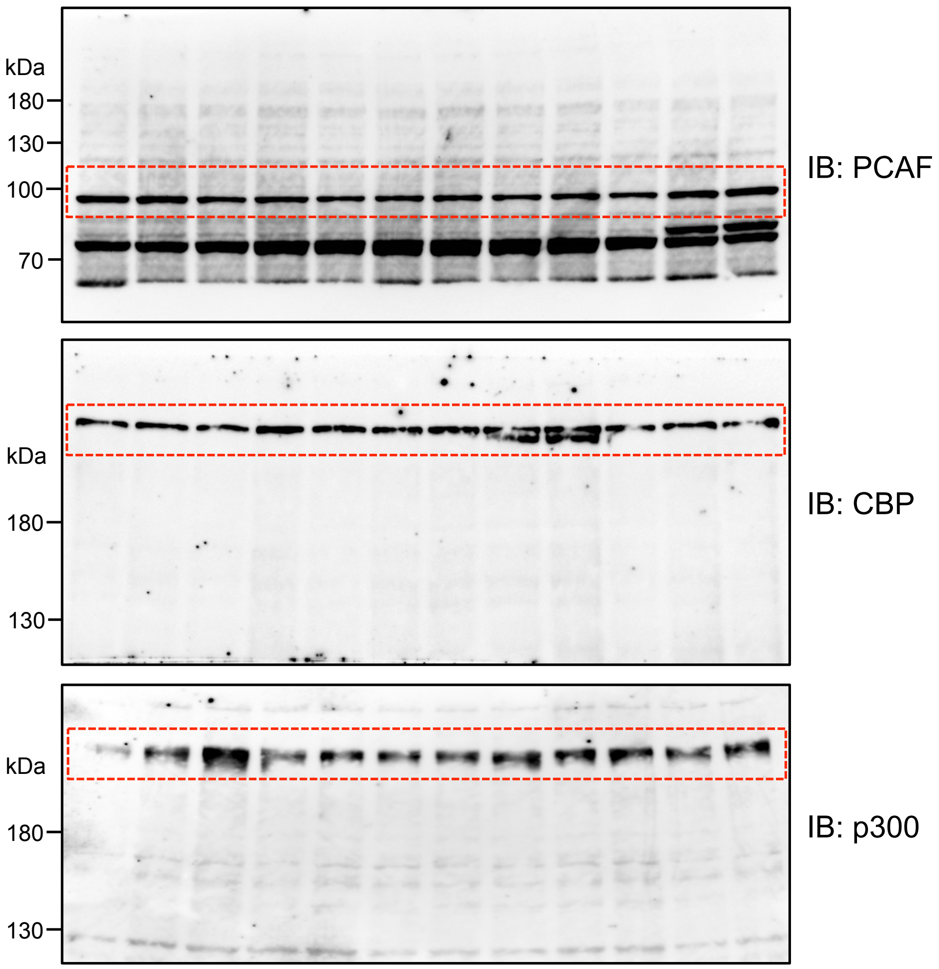
kDa
180
130
100
70
IB: PCAF
kDa
180
130
IB: CBP
kDa
180
130
IB: p300

Fig. 1d

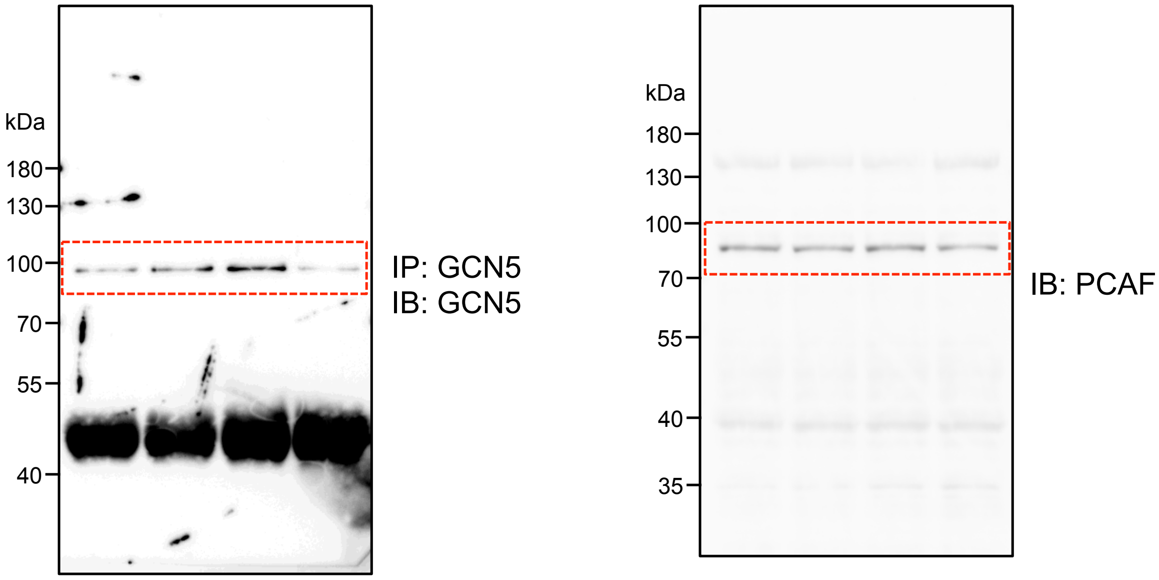
kDa
180
130
100
70
55
40
IP: GCN5
IB: GCN5
kDa
180
130
100
70
55
40
35
IB: PCAF

Fig. 2c

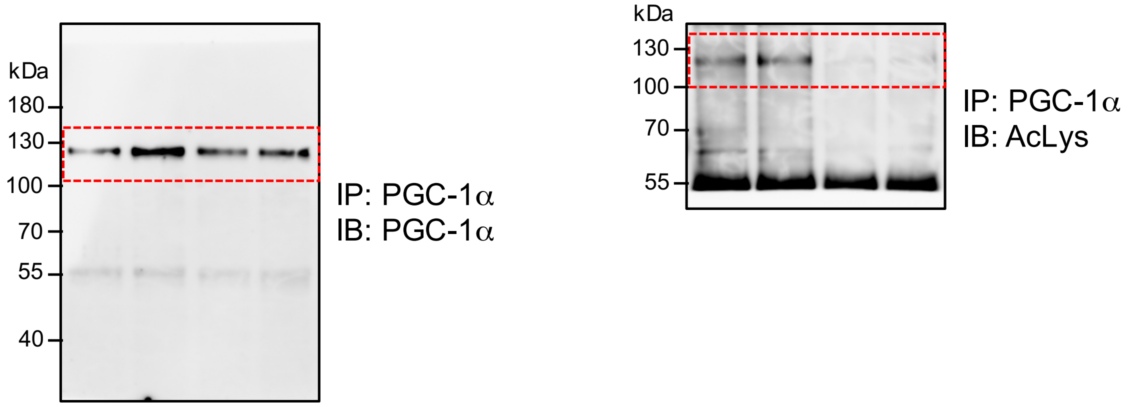

Fig. 2e

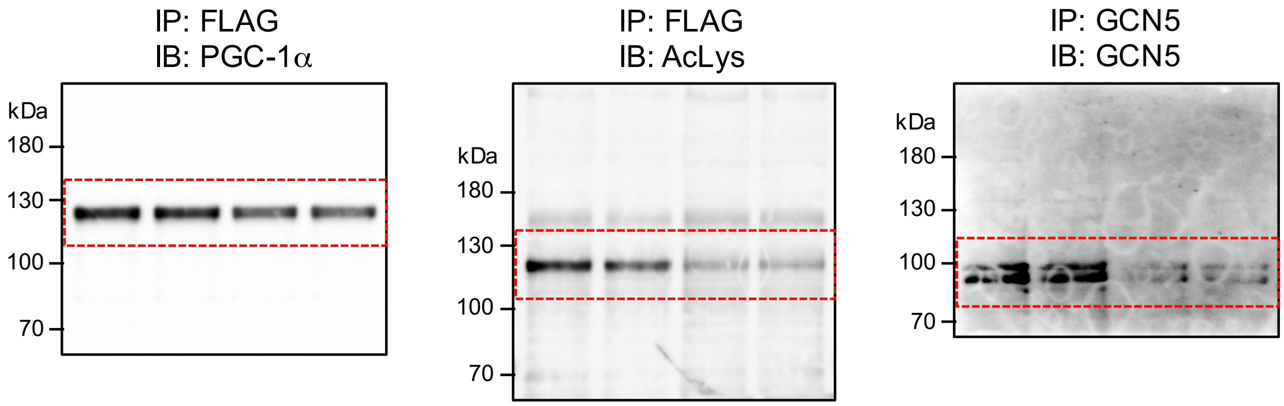

Fig. 5a

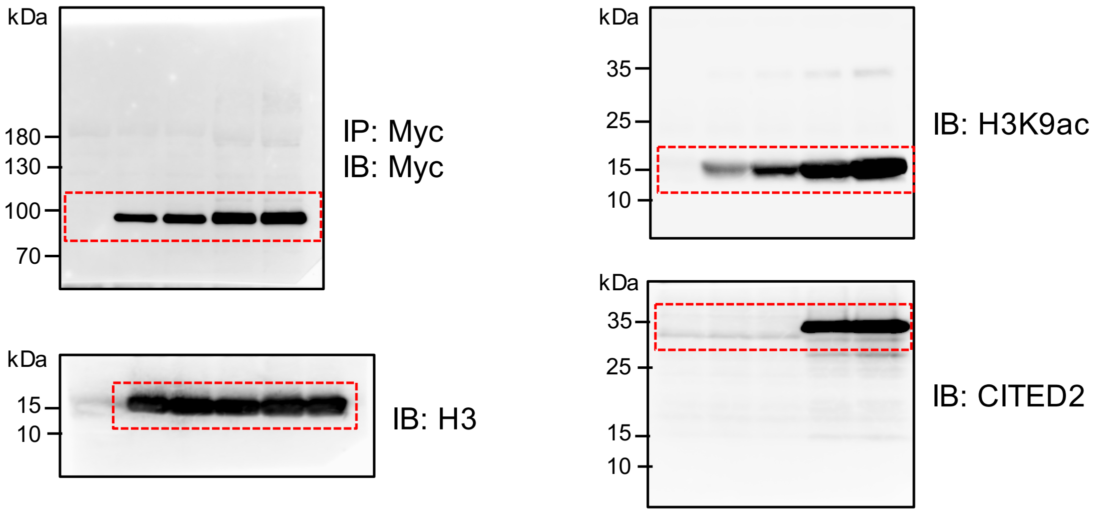

Fig. 5b

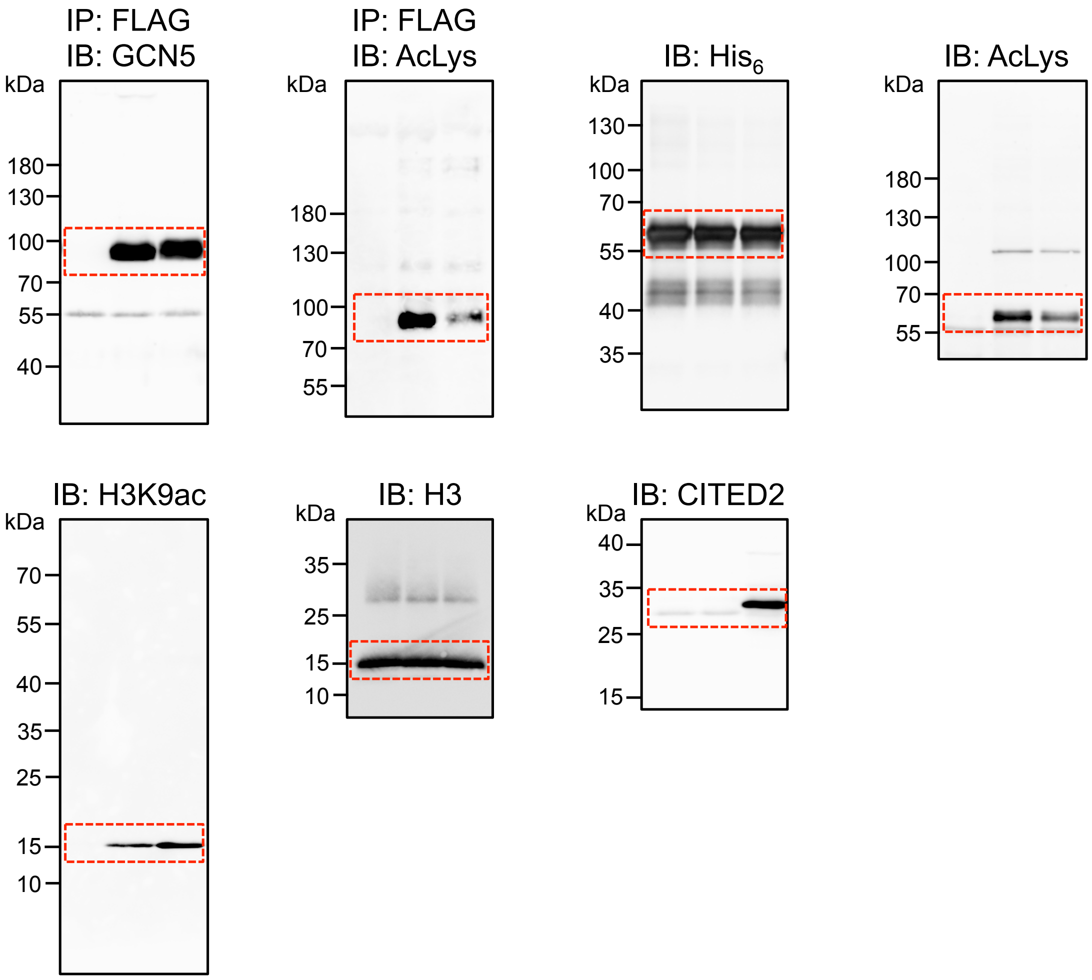
IP: FLAG
IB: GCN5
kDa
180
130
100
70
55
40
IP: FLAG
IB: AcLys
kDa
180
130
100
70
55
IB: His6
kDa
130
100
70
55
40
35
IB: AcLys
kDa
180
130
100
70
55
IB: H3K9ac
kDa
70
55
40
35
25
15
10
IB: H3
kDa
35
25
15
10
IB: CITED2
kDa
40
35
25
15

Fig. 6b

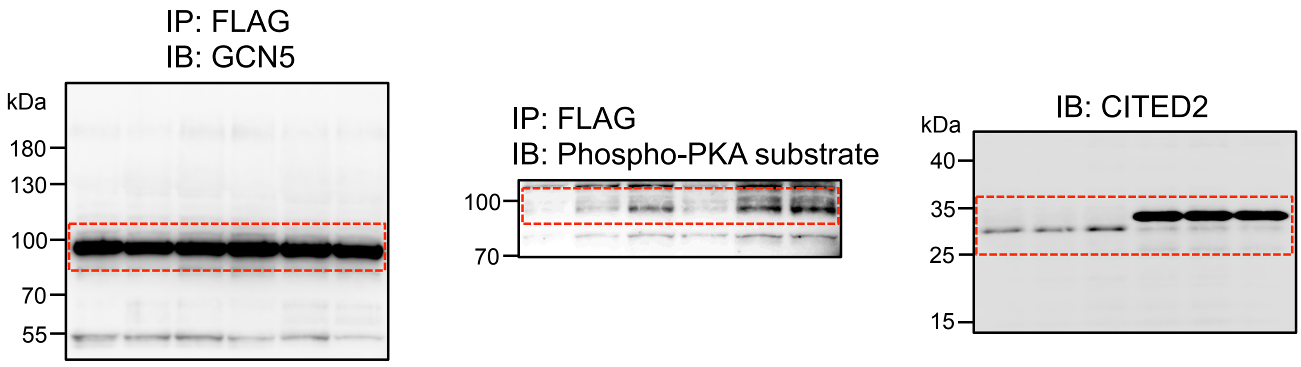
IP: FLAG
IB: GCN5
kDa
180
130
100
70
55
IP: FLAG
IB: Phospho-PKA substrate
100
70
IB: CITED2
kDa
40
35
25
15

Fig. 6c

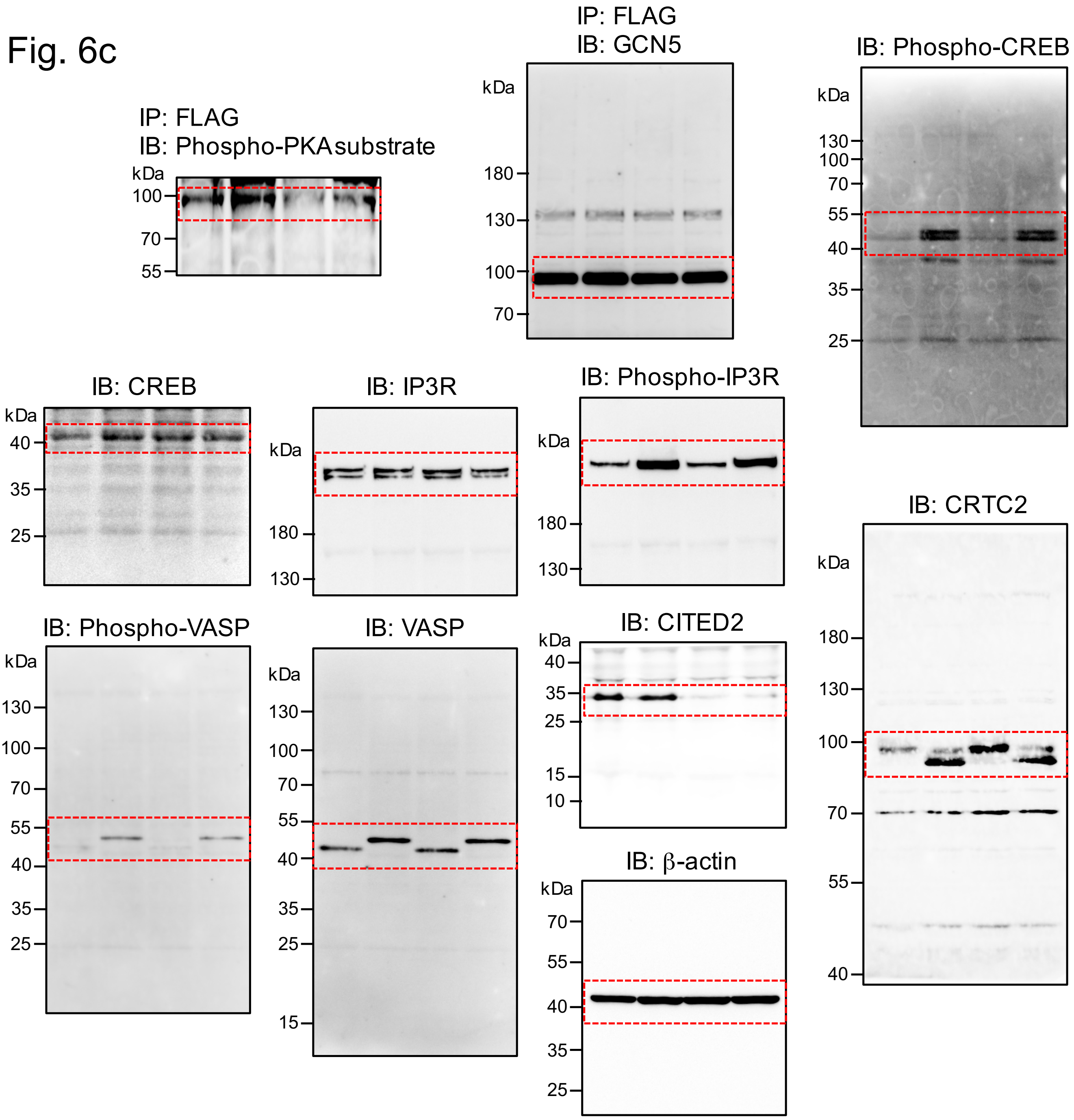
IP: FLAG
IB: Phospho-PKAsubstrate
kDa
100
70
55
IP: FLAG
IB: GCN5
kDa
180
130
100
70
IB: Phospho-CREB
kDa
130
100
70
55
40
35
25
IB: CREB
kDa
40
35
25
IB: IP3R
kDa
180
130
IB: Phospho-IP3R
kDa
180
130
IB: CRTC2
kDa
180
130
100
70
55
40
IB: Phospho-VASP
kDa
130
100
70
55
40
35
25
IB: VASP
kDa
130
100
70
55
40
35
25
15
IB: CITED2
kDa
40
35
25
15
10
IB: β-actin
kDa
70
55
40
35
25

Fig. 6d

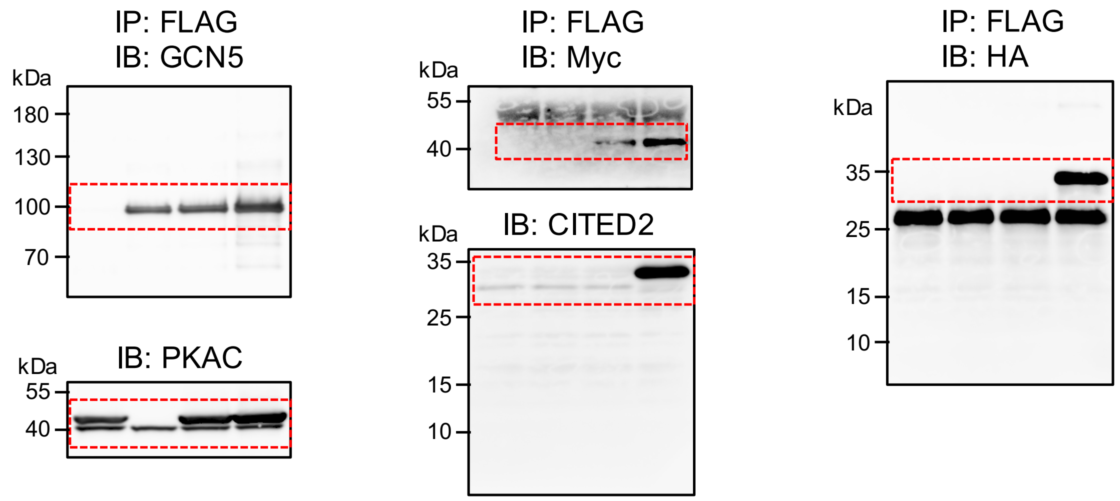
IP: FLAG
IB: GCN5
kDa
180
130
100
70
IB: PKAC
kDa
55
40
IP: FLAG
IB: Myc
kDa
55
40
IB: CITED2
kDa
35
25
15
10
IP: FLAG
IB: HA
kDa
35
25
15
10

Fig. 6e

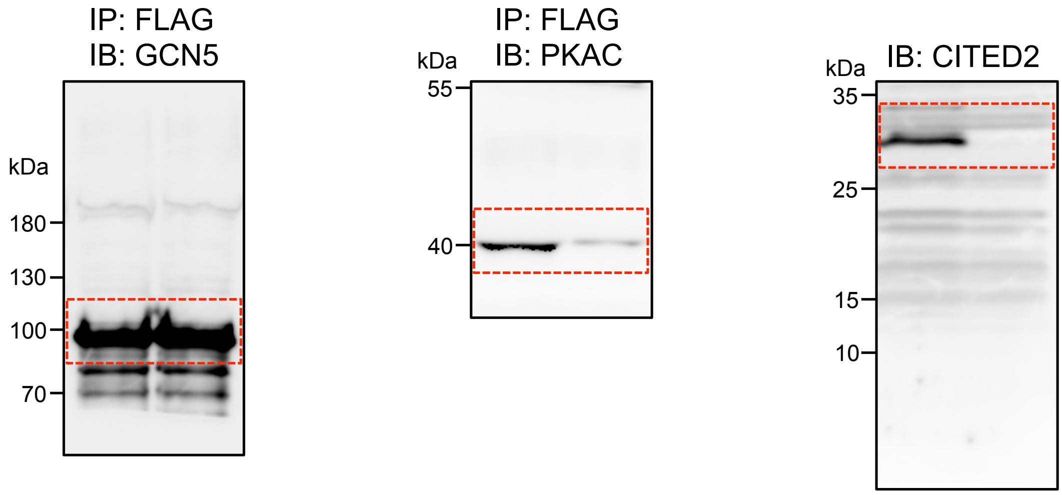

Fig. 6f

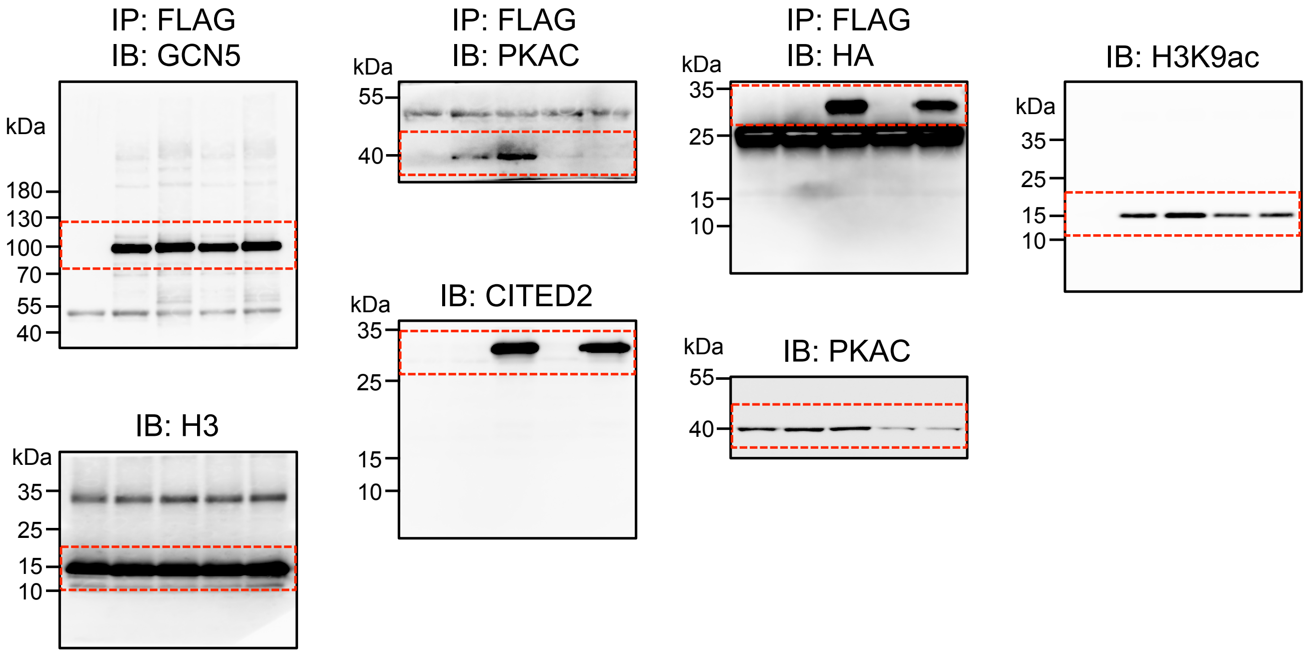

Fig. 6g

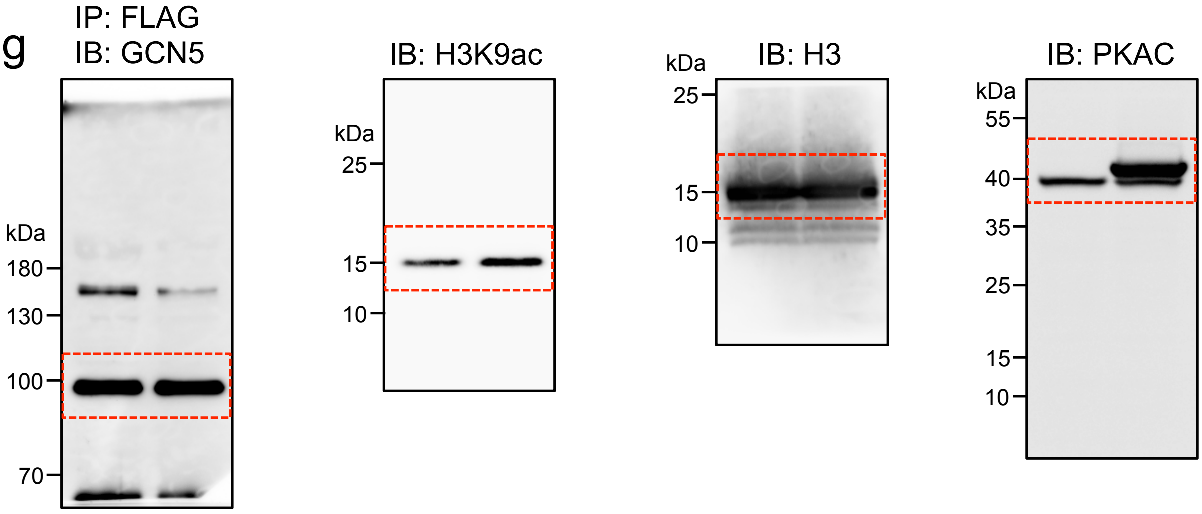

Fig. 7a

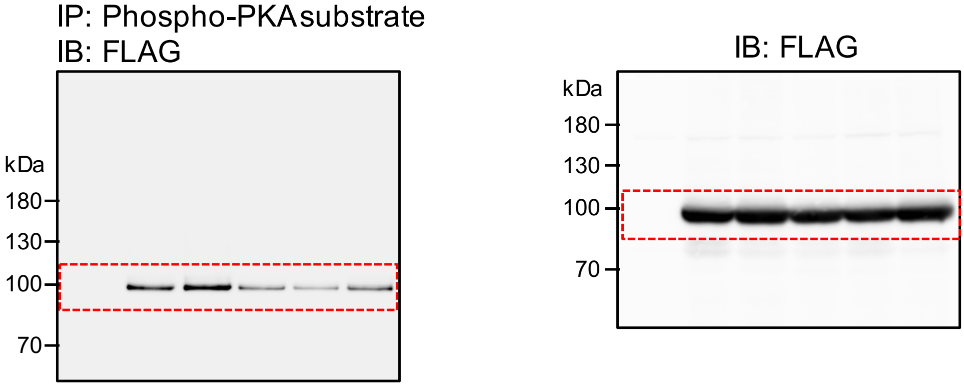

Fig. 7b

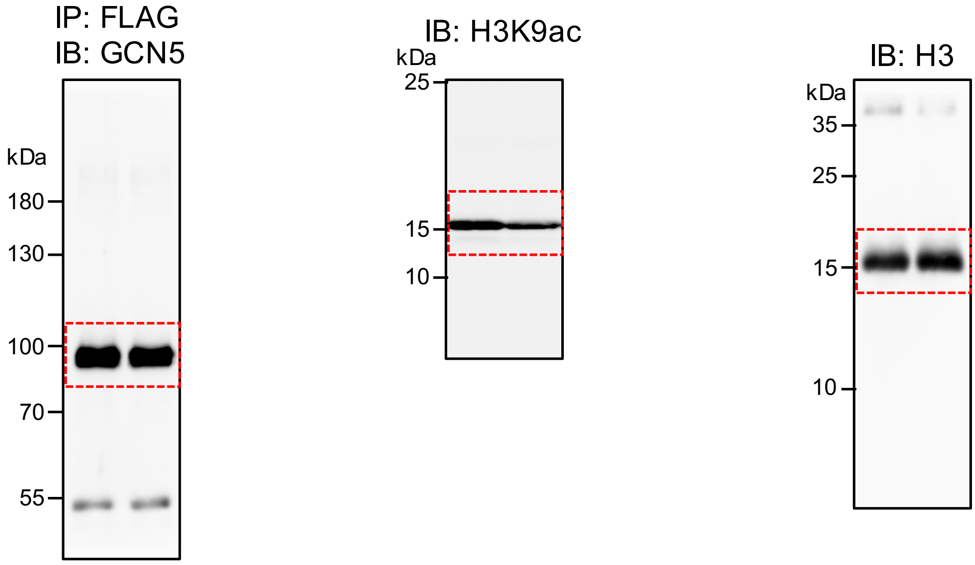

Fig. 7c

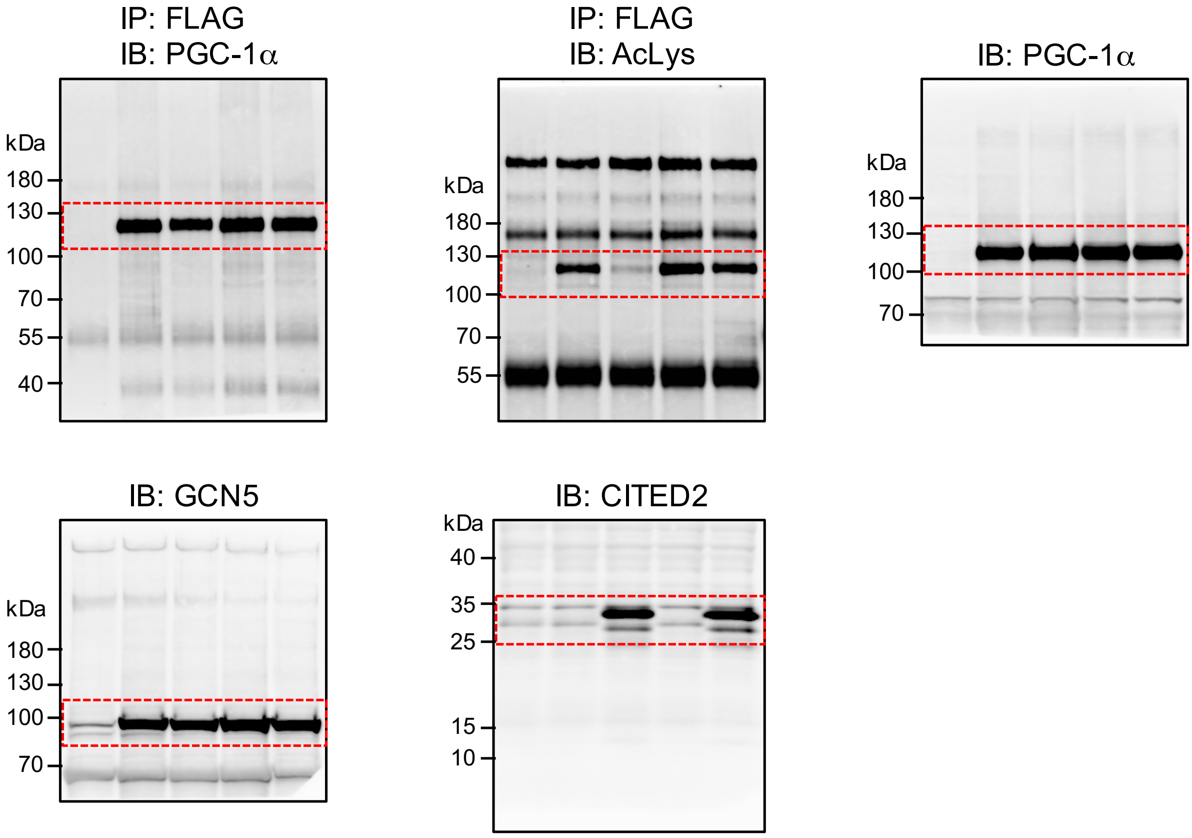

Fig. 7d

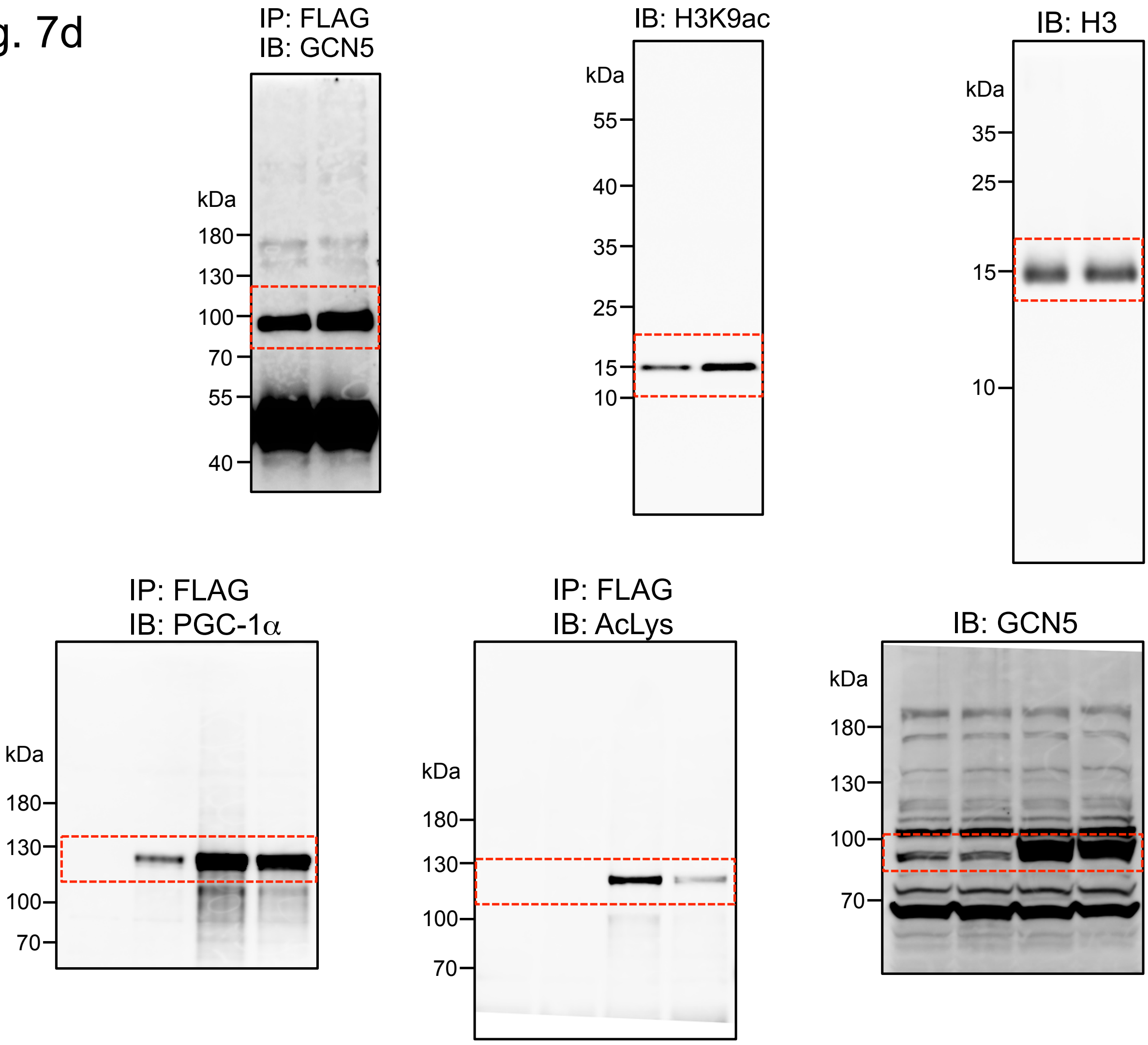
IP: FLAG
IB: GCN5
kDa
180
130
100
70
55
40
IB: H3K9ac
kDa
55
40
35
25
15
10
IB: H3
kDa
35
25
15
10
IP: FLAG
IB: PGC-1α
kDa
180
130
100
70
IP: FLAG
IB: AcLys
kDa
180
130
100
70
IB: GCN5
kDa
180
130
100
70

Fig. 7e

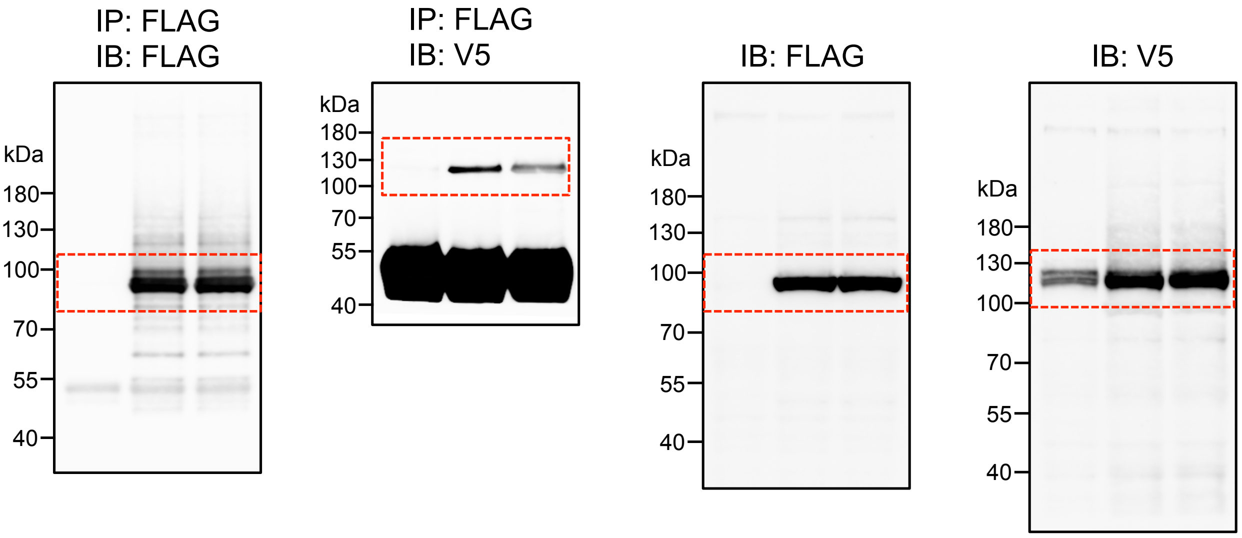
IP: FLAG
IB: FLAG
kDa
180
130
100
70
55
40
IP: FLAG
IB: V5
kDa
180
130
100
70
55
40
IB: FLAG
kDa
180
130
100
70
55
40
IB: V5
kDa
180
130
100
70
55
40

Fig. 7f

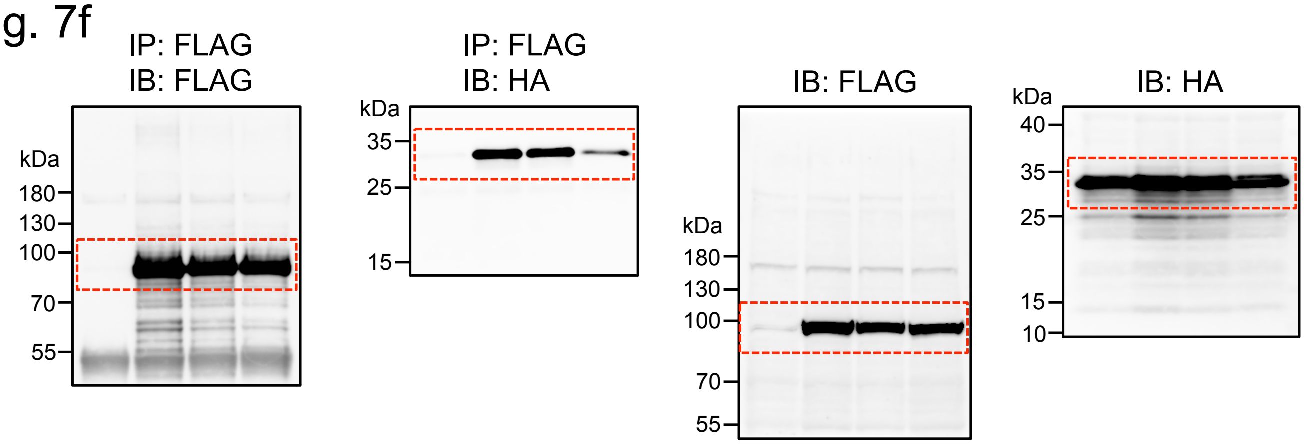

Fig. 7g

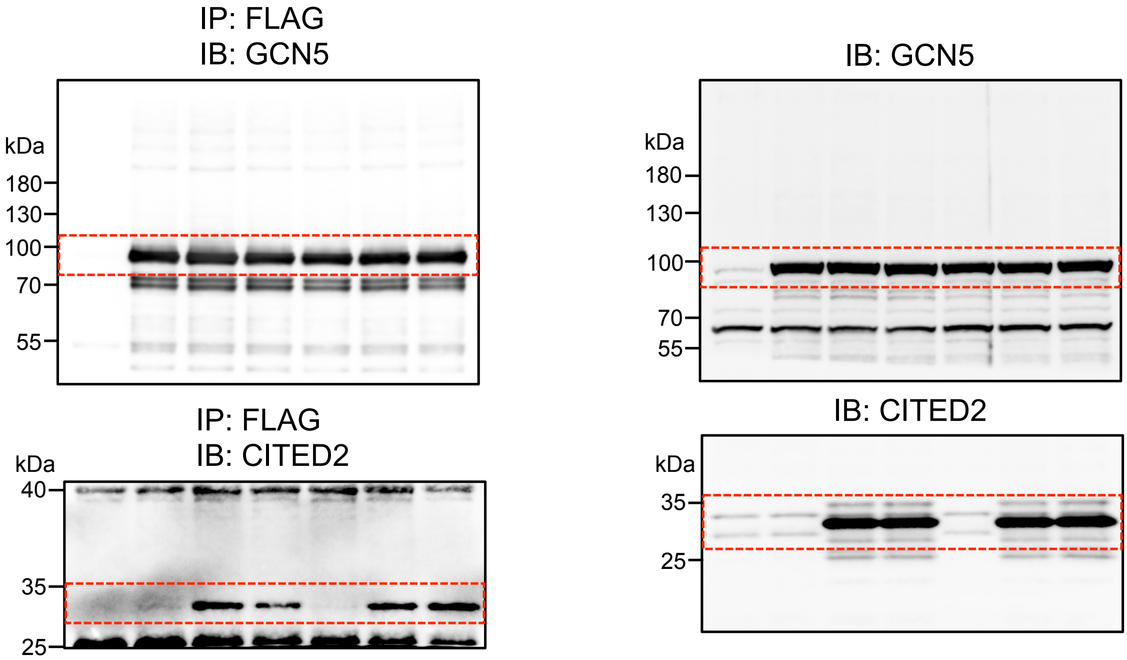

Fig. 9a

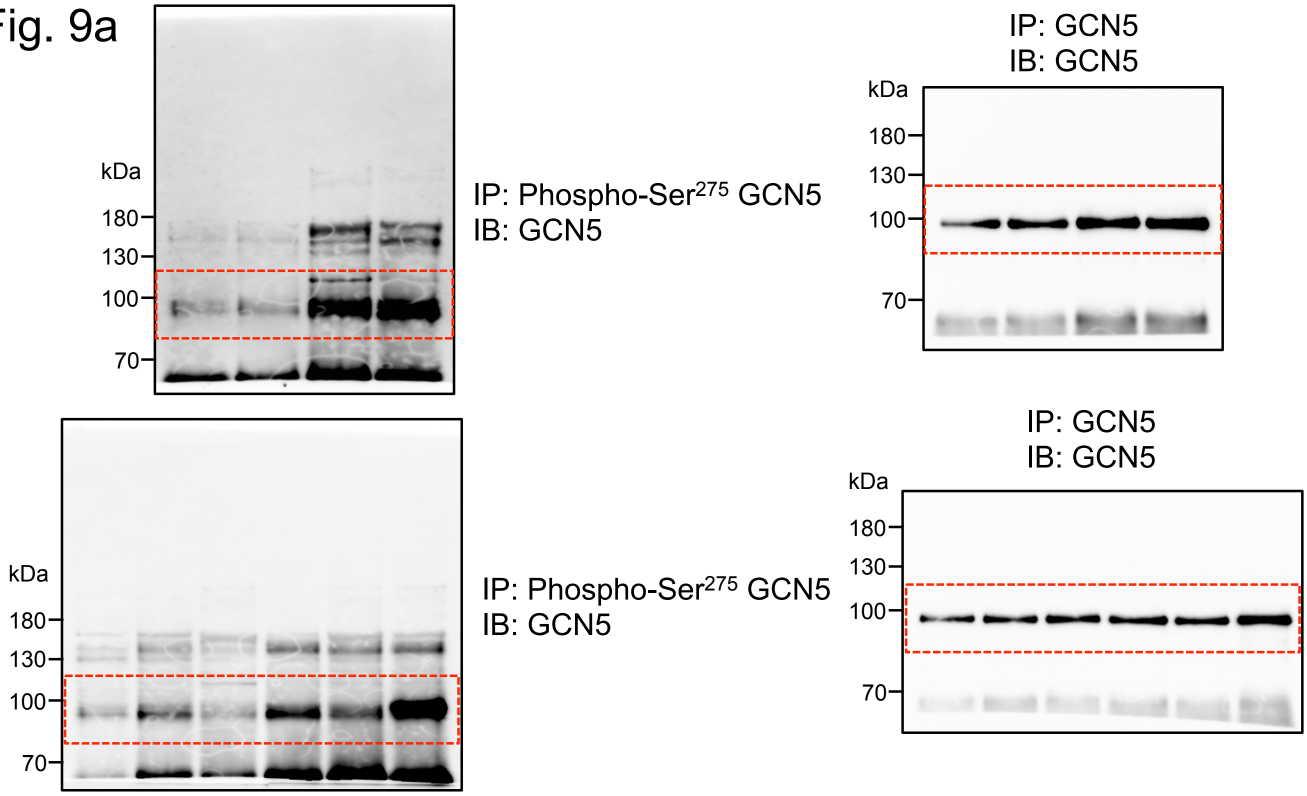

Fig. 9d

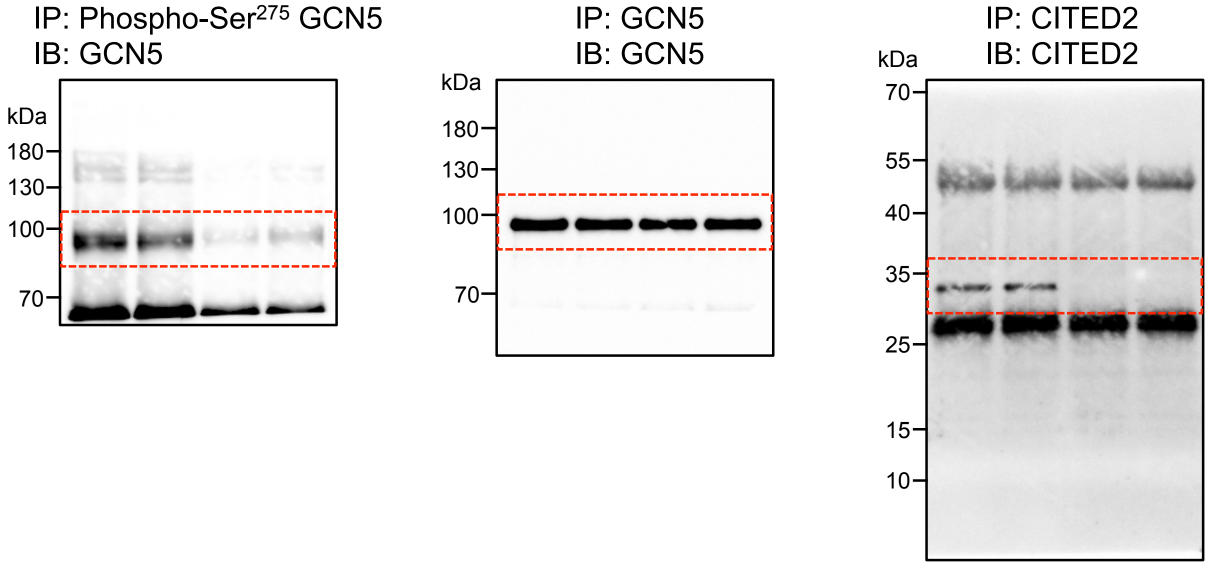

Supplementary Fig. 2a

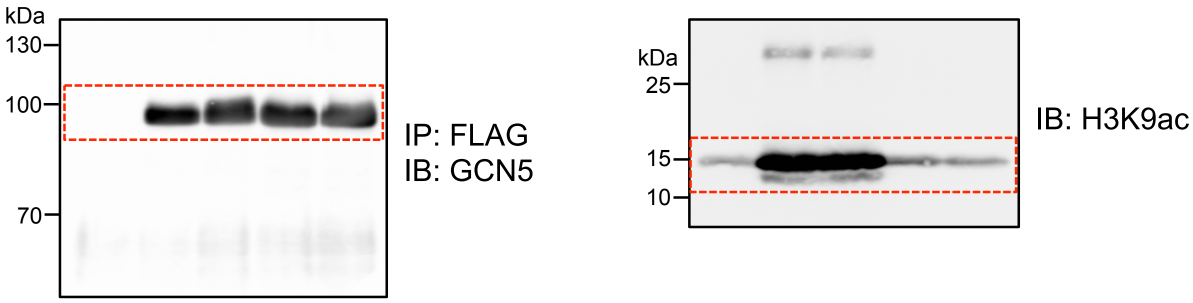

Supplementary Fig. 2b

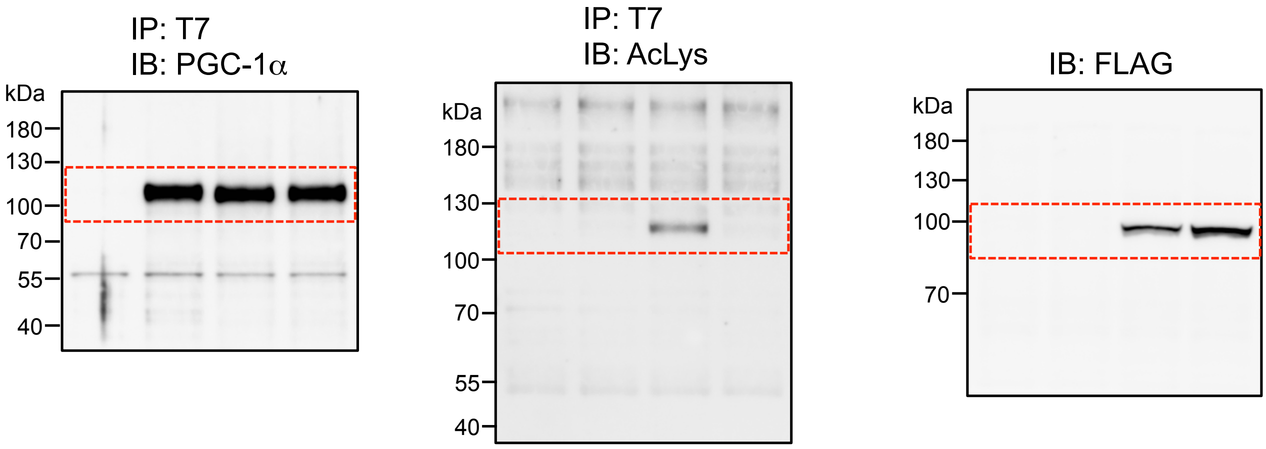

Supplementary Fig. 4a

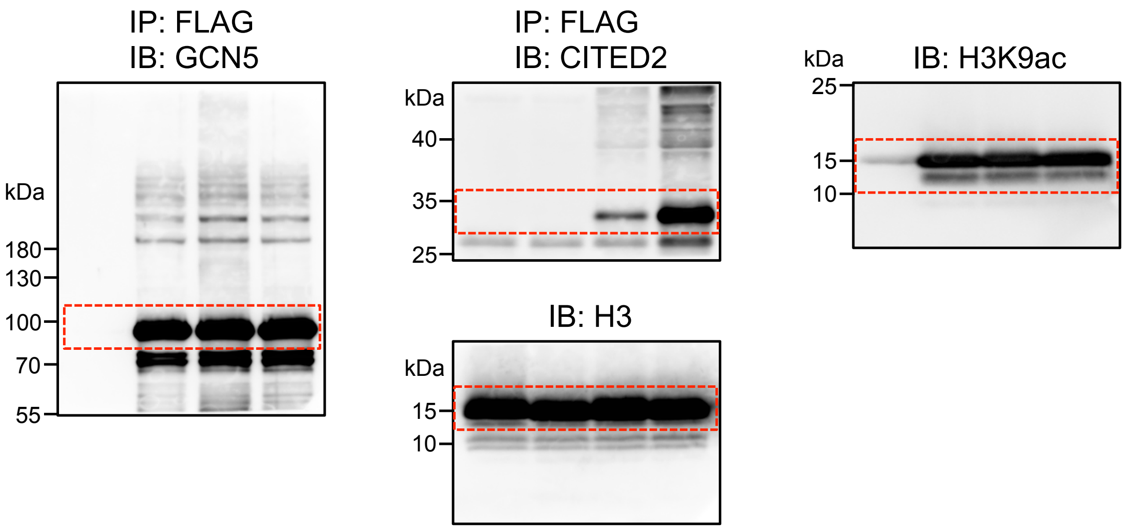

Supplementary Fig. 4b

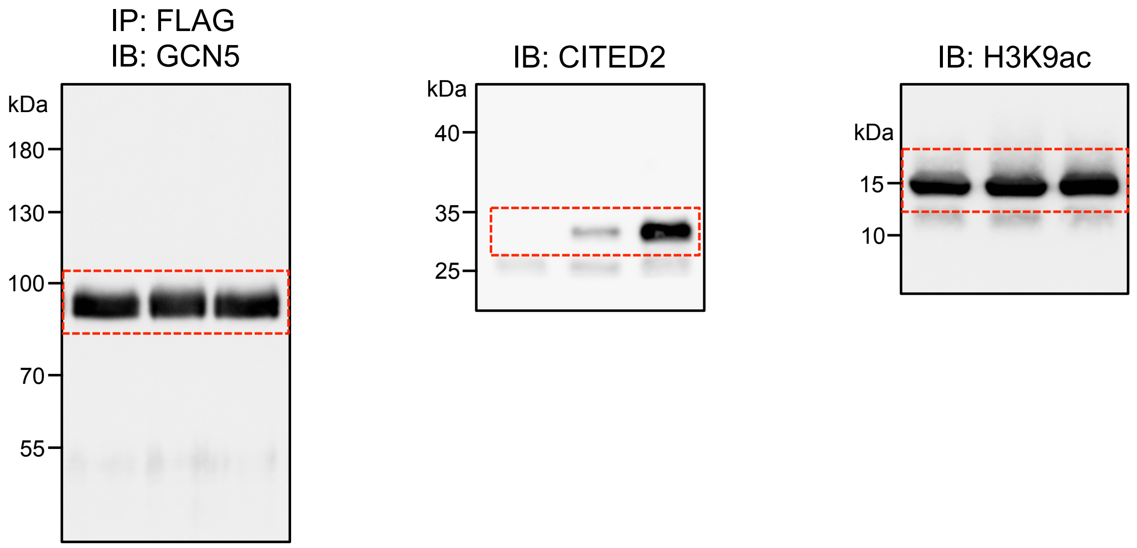

Supplementary Fig. 4d

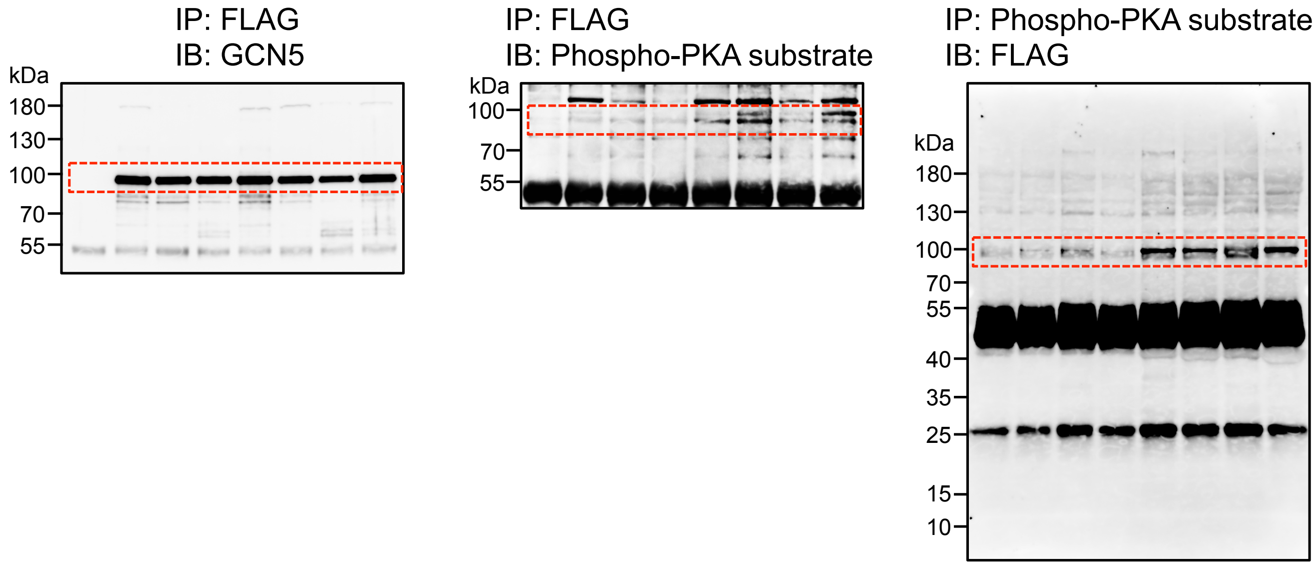

Supplementary Fig. 4e

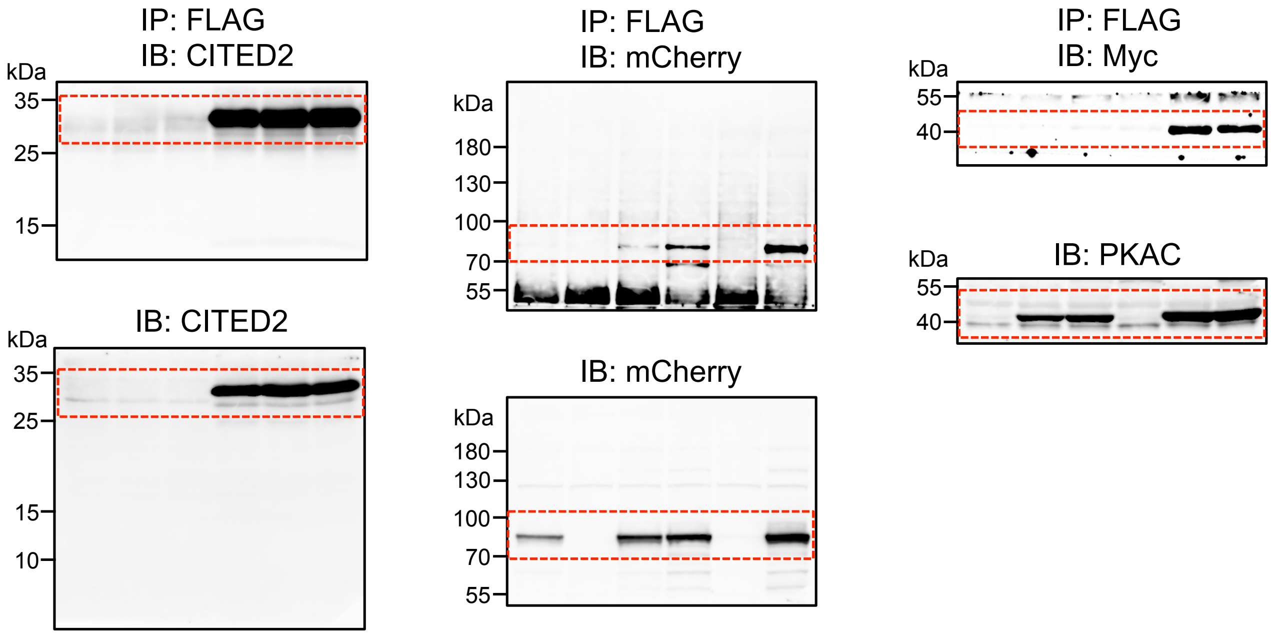

Supplementary Fig. 4f

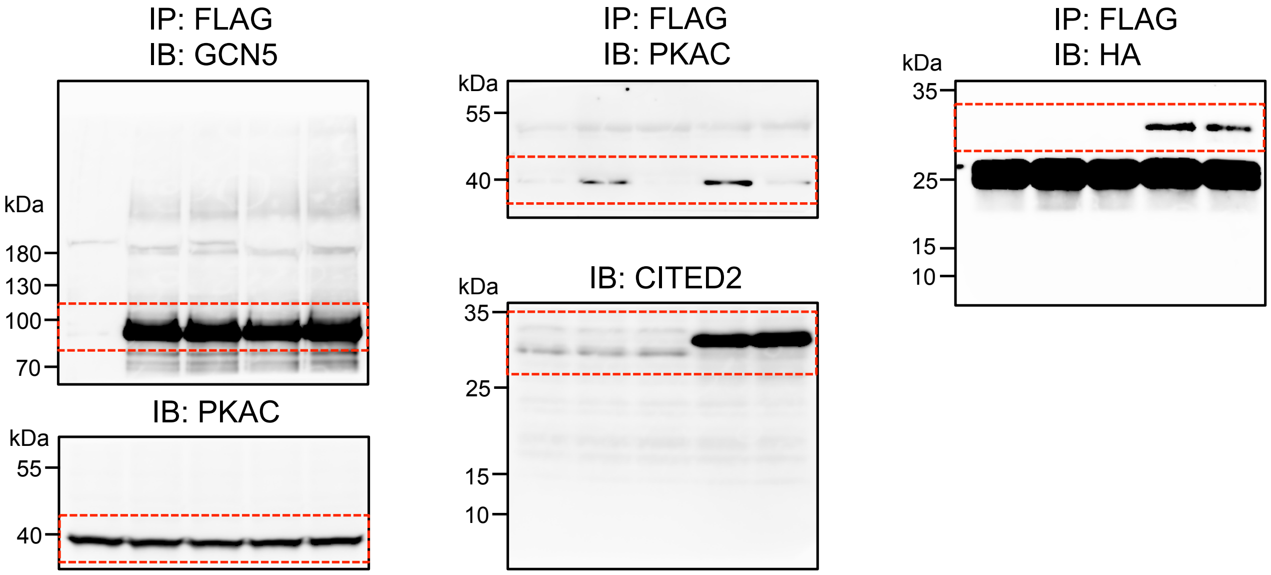

Supplementary Fig. 4g

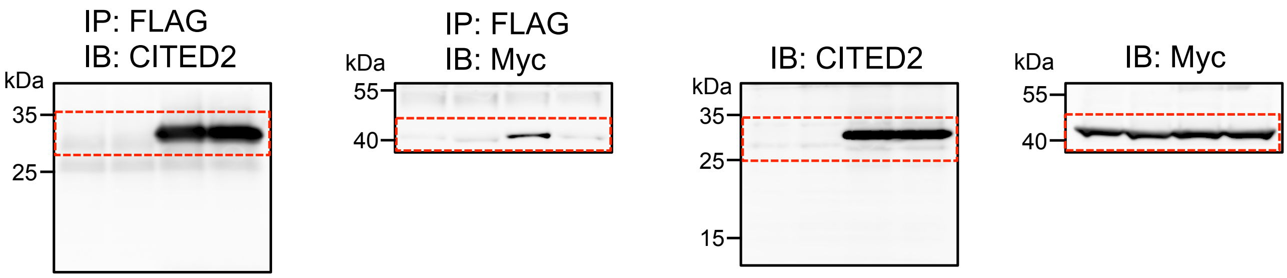

Supplementary Fig. 4h

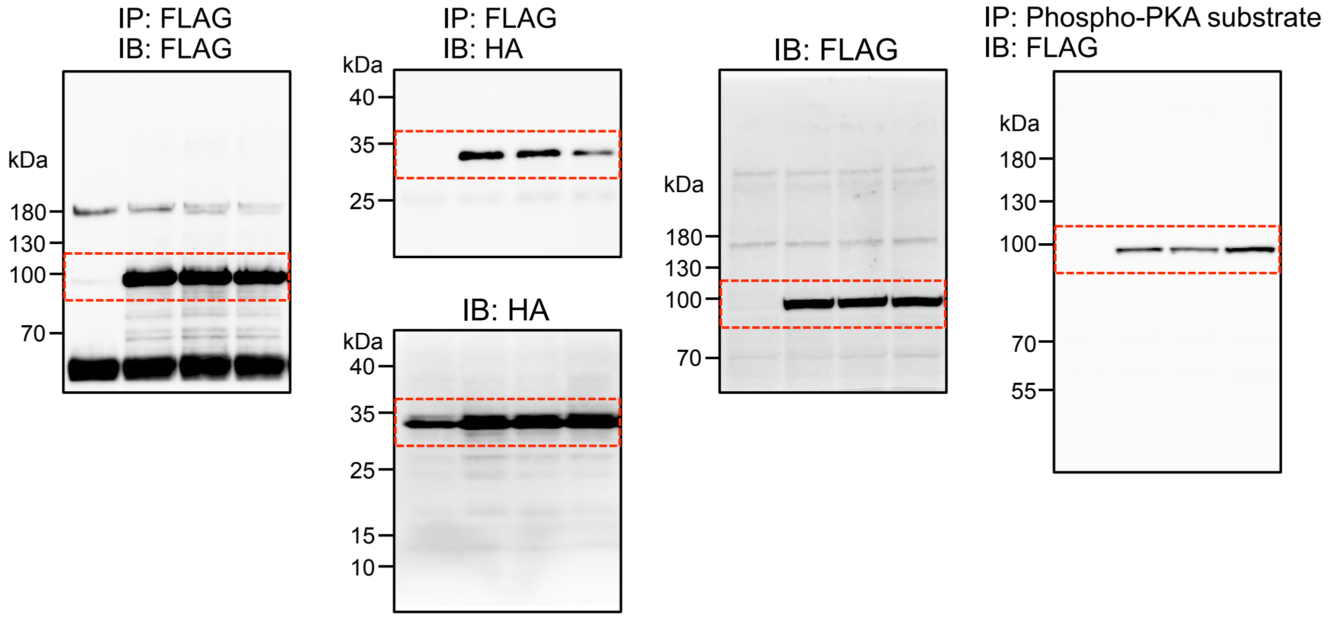

Supplementary Fig. 4i

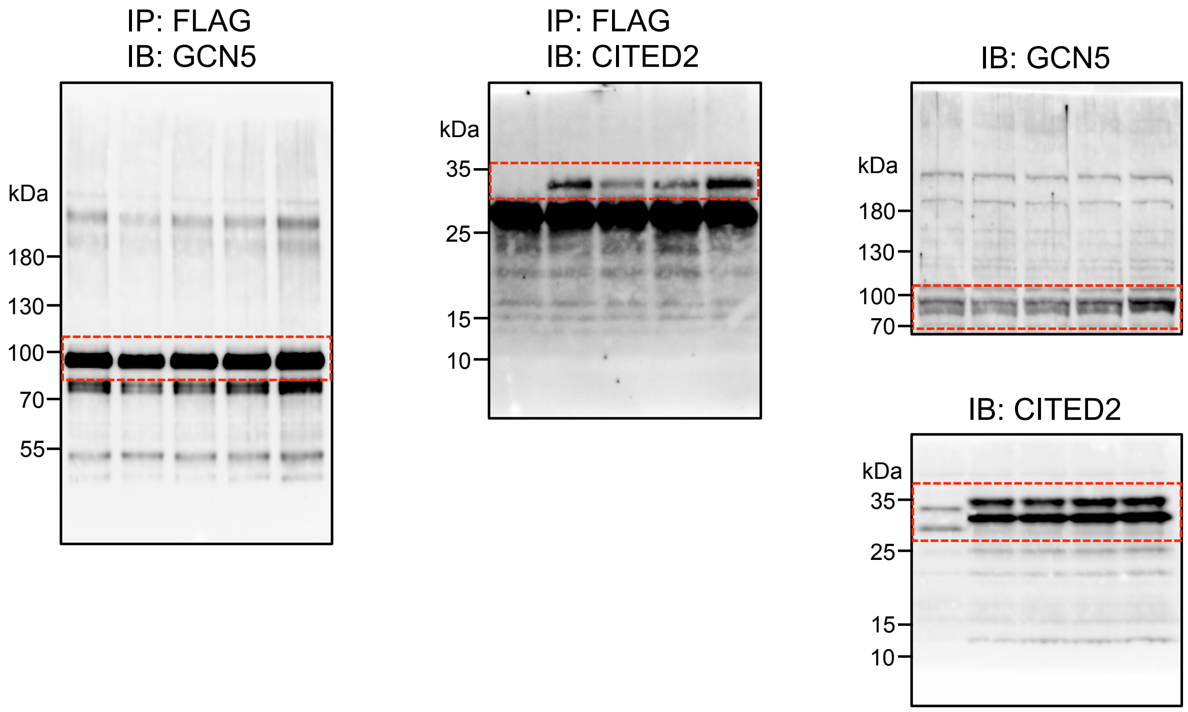

Supplementary Fig. 5b

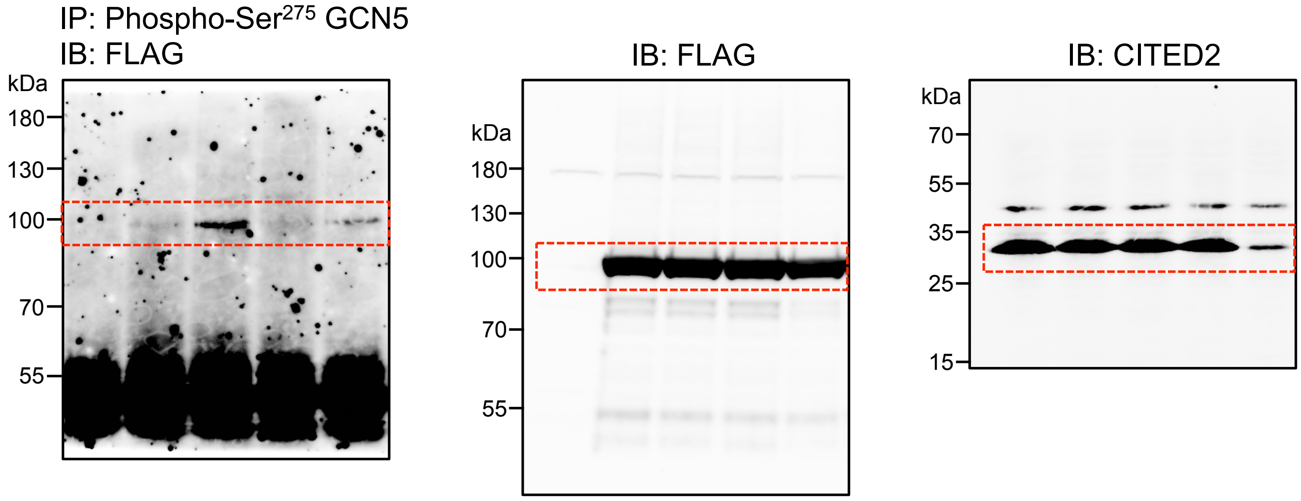

Supplementary Fig. 5c

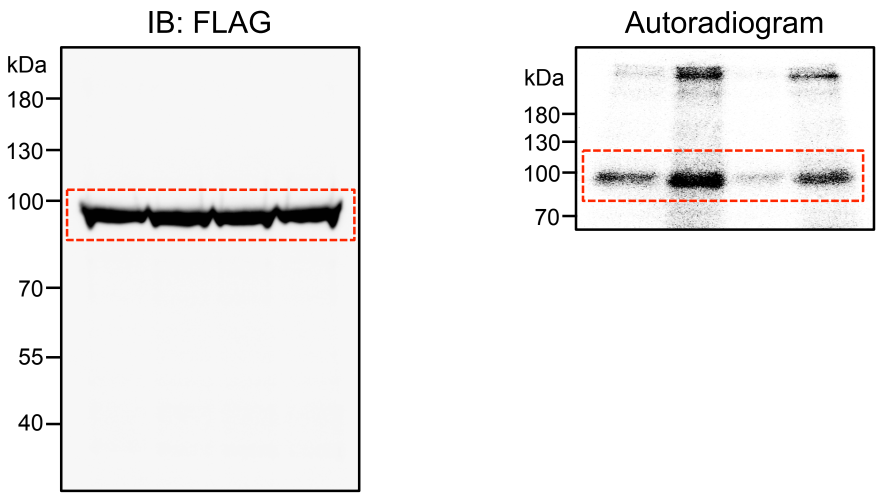

Supplementary Fig. 5d

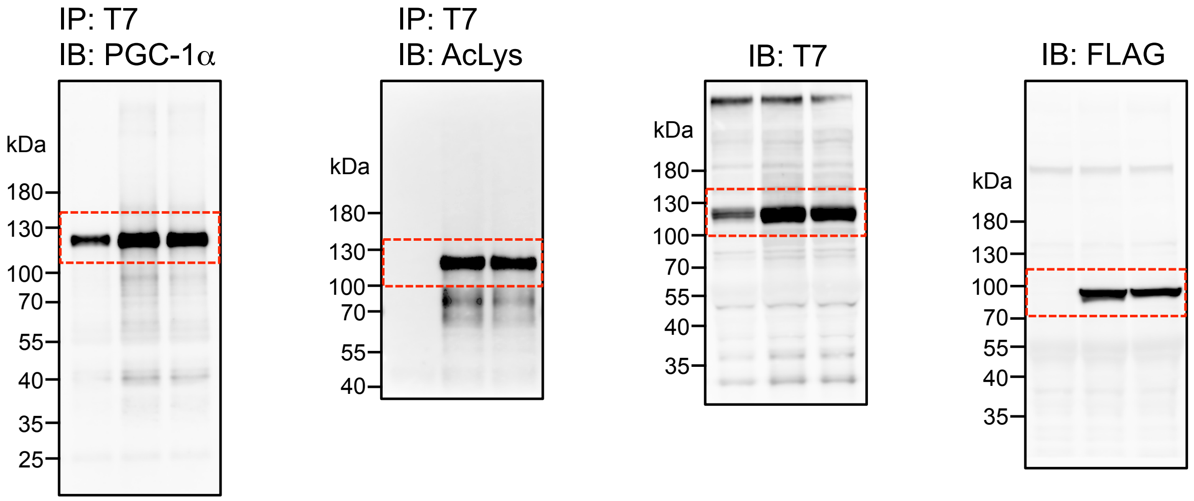

Supplementary Fig. 6e

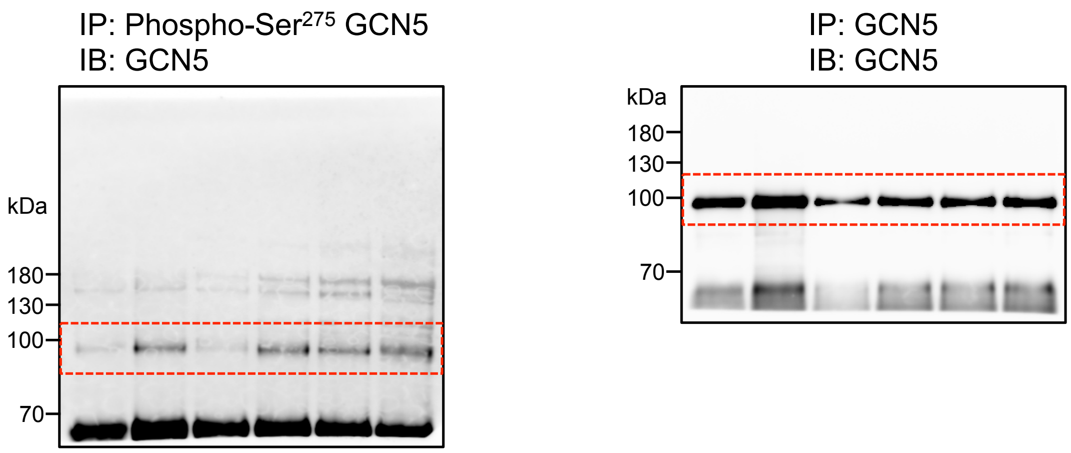

Supplementary Fig. 6f

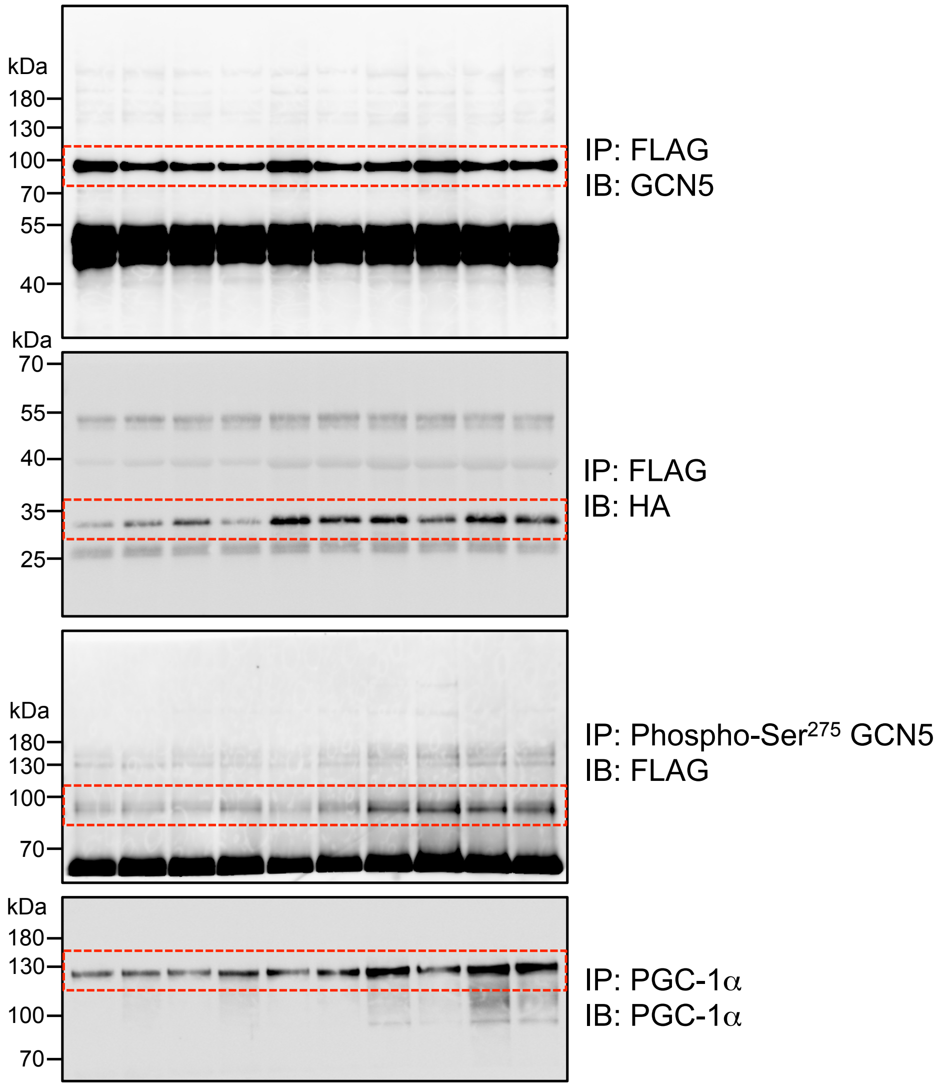

Supplementary Fig. 6f

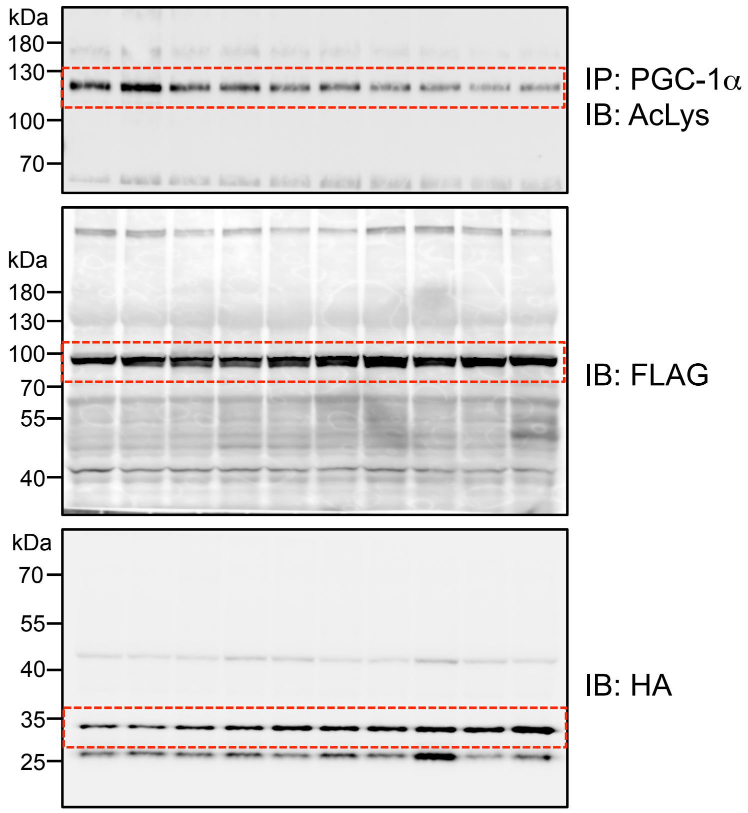

Supplementary Fig. 7a

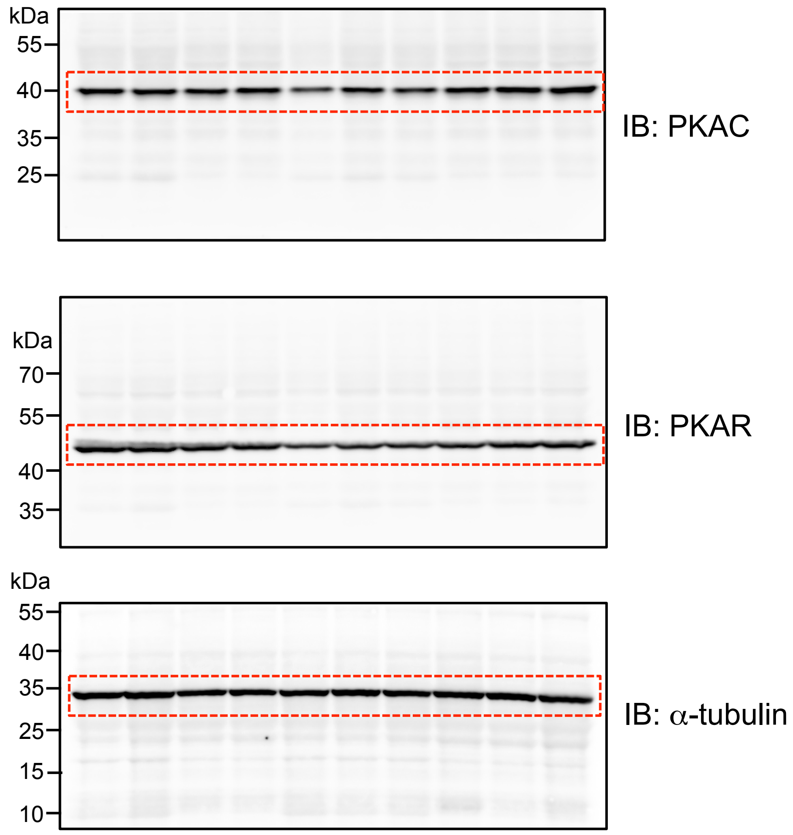

Supplementary Table 1

Primers used for qPCR

Gene	Forward	Reverse
Gcn5	5’-GTCTTCTGTGCCGTCACCTCAA-3’	5’-GCTCTTGGGCACCTTGATGTCT-3’
Cited2	5’-GCAGCTGCAGAAGCTCAACAA-3’	5’-AGTGCTGGTTTGTCCCGTTCA-3’
Ppargc1a	5’-CACAAAGACGTCCCTGCTCAGA-3’	5’-CCTTGGGGTCATTTGGTGACTC-3’
G6pc	5’-CTGTCCCGGATCTACCTTGCTG-3’	5’-AATACTTCCGGAGGCTGGCATT-3’
Pck1	5’-CCTGGAAGAACAAGGAGTGG-3’	5’-AGGGTCAATAATGGGGCACT-3’
Gapdh	5’-TCCCAGAGCTGAACGGGAAG-3’	5’-CCCAAGATGCCCTTCAGTGG-3’
Hmgcr	5’-GCAGCTCAGCCATTTTGCCAGA-3’	5’-AGCGTGAACAAGGACCAAGCCT-3’

Primers used for ChIP-qPCR

Location	Forward	Reverse
G6pc promoter	5'-GCGATCAGGCTGTTTTTGTGT-3'	5'-TCCAGCCCTGATCTTTGGACT-3'
Pck1 promoter_1	5'-TGAGGCCTCCCAACATTCATT-3	5'-TTGGATCATAGCCATGGTCAGC-3'
Pck1 promoter_2*	5'-GCTGAATTTCCTTCTCATGAC-3'	5'-TCATATGTTGCTGGCTGCAC-3'
Gapdh promoter	5'-TGTGCAGTGCCAGGTGAAAATC-3	5'-CTTCGCACCAGCATCCCTAGAC-3'
Hmgcr promoter	5'-CTCTGCCGTGGTGAGAGATGGT-3'	5'-ACGTCACGAACGGTCTCCCTAA-3'

*For ChIP of HNF-4α, FLAG-PGC-1α, or FLAG-FoxO1
